# Binucleate germ cells in *Caenorhabditis elegans* are removed by physiological apoptosis

**DOI:** 10.1371/journal.pgen.1007417

**Published:** 2018-07-19

**Authors:** Stephan A. Raiders, Michael D. Eastwood, Meghan Bacher, James R. Priess

**Affiliations:** 1 Fred Hutchinson Cancer Research Center, Seattle, Washington, United States of America; 2 Molecular and Cellular Biology Program, University of Washington, Seattle, Washington, United States of America; 3 Department of Biology, University of Washington, Seattle, Washington, United States of America; University of California San Diego, UNITED STATES

## Abstract

Cell death plays a major role during *C*. *elegans* oogenesis, where over half of the oogenic germ cells die in a process termed physiological apoptosis. How germ cells are selected for physiological apoptosis, or instead become oocytes, is not understood. Most oocytes produce viable embryos when apoptosis is blocked, suggesting that physiological apoptosis does not function to cull defective germ cells. Instead, cells targeted for apoptosis may function as nurse cells; the germline is syncytial, and all germ cells appear to contribute cytoplasm to developing oocytes. *C*. *elegans* has been a leading model for the genetics and molecular biology of apoptosis and phagocytosis, but comparatively few studies have examined the cell biology of apoptotic cells. We used live imaging to identify and examine pre-apoptotic germ cells in the adult gonad. After initiating apoptosis, germ cells selectively export their mitochondria into the shared pool of syncytial cytoplasm; this transport appears to use the microtubule motor kinesin. The apoptotic cells then shrink as they expel most of their remaining cytoplasm, and close off from the syncytium. Shortly thereafter the apoptotic cells restructure their microtubule and actin cytoskeletons, possibly to maintain cell integrity; the microtubules form a novel, cortical array of stabilized microtubules, and actin and cofilin organize into giant cofilin-actin rods. We discovered that some apoptotic germ cells are binucleate; the binucleate germ cells can develop into binucleate oocytes in apoptosis-defective strains, and appear capable of producing triploid offspring. Our results suggest that the nuclear layer of the germline syncytium becomes folded during mitosis and growth, and that binucleate cells arise as the layer unfolds or everts; all of the binucleate cells are subsequently removed by apoptosis. These results show that physiological apoptosis targets at least two distinct populations of germ cells, and that the apoptosis machinery efficiently recognizes cells with two nuclei.

## Author summary

Many germ cells die by apoptosis during the development of animal oocytes, including more than half of all germ cells in the model system *C*. *elegans*. How individual germ cells are selected for apoptosis, or survival, is not known. Here we study the cell biology of apoptosis. The *C*. *elegans* gonad is a syncytium, with nearly 1000 germ “cells” connected to a shared, core cytoplasm. Once apoptosis is initiated, germ cells selectively transport their mitochondria into the gonad core, apparently using the microtubule motor protein kinesin. The apoptotic cells next constrict, expelling most of their remaining cytoplasm into the core, and close off from the gonad core. The microtubule and actin cytoskeletons are remodeled and stabilized, presumably to maintain the integrity of the dying cell. The apoptotic cells form giant cofilin-actin rods, similar to rods described in stressed cultured cells and in human myopathies and neuropathies such as Alzheimer’s and Huntington’s disease. We show that some germ cells are binucleate; these cells appear to form during germline morphogenesis, and are removed by apoptosis. These results demonstrate heterogeneity between oogenic germ cells, and show that the apoptosis machinery efficiently recognizes and removes cells with two nuclei.

## Introduction

Apoptosis is a universal feature of animal development, and allows the controlled demolition of damaged or unnecessary cells and the subsequent removal of those cells by phagocytes [1]. The major molecular components of the apoptosis and corpse-removal pathways are conserved evolutionarily, and many were identified through pioneering genetic studies on *C*. *elegans* [2] [3]. About 17% of embryonic cells undergo apoptosis in *C*. *elegans* [4], as do more than 50% of adult germ cells [5–7]. *C*. *elegans* embryogenesis occurs through a nearly invariant cell lineage, and the vast majority of embryonic deaths target specific, predictable cells within the lineage [4]. By contrast, the germline develops from a dynamic, self-renewing population of stem cells that is maintained as a niche [8]. DNA damage and other stresses can induce germ cell death, but the deaths observed in normal development, termed physiological apoptosis, do not involve the known damage or stress pathways [9] [5].

Apoptosis involves three distinct phases of induction, execution, and clearance. A key step is the activation of caspase-family proteases, such as *C*. *elegans* CED-3. Mammalian cells and *Drosophila* can have specialized initiator and executioner caspases [10, 11], but CED-3 appears to be the main effector of apoptosis in *C*. *elegans* [12]. During the execution phase, most apoptotic cells undergo chromatin condensation and reorganize their cytoskeleton. Apoptotic cells can become rounded and lose adhesion to surrounding tissues as they develop a contractile, cortical actomyosin network. The remodeled actin cytoskeleton can function in the formation of membrane blebs and, in some cells, the fragmentation of the cell into apoptotic bodies [13]. Microtubules are often depolymerized during the early stages of apoptosis, but recent studies have shown some apoptotic cells reorganize their microtubules (MTs) into a novel array, called the apoptotic MT network [14]. Similarly, cytoplasmic organelles such as the Golgi apparatus and mitochondria become reorganized during apoptosis [15]. For example, mitochondria can form aggregates and/or fragment during apoptosis in some systems, including somatic cells in *C*. *elegans* [16]. Germ cells in *C*. *elegans* become round and develop condensed chromatin during apoptosis [5]. However, other possible aspects of cell biology have received little attention, possibly because the clearance phase occurs almost immediately: *C*. *elegans* embryos contain diverse cells that are capable of engulfing and degrading apoptotic cells, and nearly all germ cells in the adult gonad are in direct contact with phagocytic somatic cells, called sheath cells [17] [9]. Apoptotic cells in *C*. *elegans* and other systems express surface “eat me” signals such as phosphatidylserine that promote their recognition by phagocytic cells [18]. Phagocytic cells engulf or enclose the apoptotic cell in a vesicular phagosome, which can fuse with a lysosome to become a phagolysosome [19].

Physiological apoptosis in *C*. *elegans* is associated with oogenesis. Apoptosis does not occur in male gonads, or in larval hermaphrodites that produce sperm before switching as adults to oogenesis [9]. Apoptosis has a major role in oogenesis in many animals, including mammals where the majority of germ cells die [20] [21]. Germ cells targeted for apoptosis might be defective or less able to compete for survival [22], or might function as specialized nurse cells as in *Drosophila* [23]. The *C*. *elegans* germline is described by convention as consisting of germ cells; however, the germline is a syncytium, as in many animals from *Drosophila* to mammals [24]. In *C*. *elegans*, this syncytial organization presumably allows all germ cells to contribute to the biosynthesis of oocyte materials [25]. Thus, if an individual germ cell undergoes apoptosis instead of becoming an oocyte, it can be considered to have functioned as a nurse cell [5].

Physiological apoptosis is thought to be specific for late pachytene germ cells, shortly before they enlarge in size, and to occur near a morphological bend in the gonad called the loop [5]. The MAP kinase pathway is thought to play a role in cell death, and MAP kinase activation peaks just before the loop region [5, 26, 27]. It is not known how *C*. *elegans* germ cells are selected for physiological apoptosis, or are instead designated to become oocytes; no cytological or molecular differences have been reported between the two populations of germ cells in normal development. Germ cells fated for apoptosis do not appear to have defective chromosomes, as about 88% of the eggs produced by apoptosis-defective *ced-3* or *ced-4* mutants hatch and develop [28]. *C*. *elegans* gonads contain large quantities of the retrotransposon Cer1 when adult hermaphrodites are cultured at low temperatures, and variable numbers of Cer1 capsids enter oogonia in the same region where germ cells undergo apoptosis [29]. However, it is not known whether Cer1 contributes to physiological apoptosis at low temperature. Computational models of germ cell development are consistent with a random specification of apoptotic cells, but also support a size-based selection mechanism [30].

We are interested in identifying factors that induce physiological apoptosis, or that promote oocyte development. In the present study we examine the cell biology of germ cell apoptosis, with a focus on apoptosis in engulfment-defective mutants. Although apoptotic cells are rapidly engulfed and cleared by phagocytosis in wild-type animals, they can persist for days in engulfment-defective mutants [31]. Thus, mechanisms likely exist that maintain the integrity of apoptotic cells. We show here that the execution phase of apoptosis involves the selective removal of all or most of the mitochondria, followed by the shrinkage and closure of the cell. The closed cells undergo major changes in their cytoskeletal organization, and we propose that these changes contribute to the stability of the cell corpses. We demonstrate that the population of germ cells removed by physiological apoptosis includes cells with two nuclei. These binucleate cells appear to form during gonad morphogenesis; cell proliferation creates folds in the germline syncytium, and binucleate cells occur during the eversion of the folds. Binucleate oocytes are present in apoptosis-defective *ced-3* mutants, and appear to have the potential to become viable, triploid progeny. We conclude that there are at least two types of germ cells that undergo physiological apoptosis, and that the apoptotic machinery effectively recognizes and removes germ cells that have two nuclei, but possibly no other defects.

## Results

### Background

**Fig 1:**
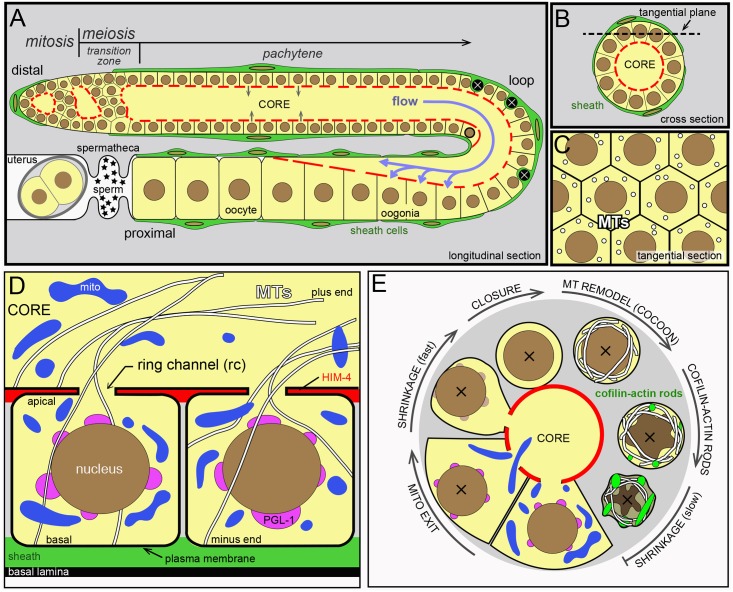
Diagram of the *C*. *elegans* gonad. (A-C) Images shown in this report are longitudinal optical sections (A), cross sections (B), or tangential sections (C). All images are oriented with distal left and proximal right. The gonad contains germ cells and somatic sheath cells (green), and is surrounded by a basal lamina (see panel D). Sheath cells cover most of the gonad, except for a region near the distal end. Germ cells are born in the mitotic zone and enter meiosis in the transition zone, where chromosomes pair. Germ cells move in the proximal direction as they progress through various stages of meiosis, and slowly and intermittently contribute cytoplasm to the core (grey arrows). Cells nearing the gonad loop undergo apoptosis (black cells), or rapidly intercalate and enlarge as they take up core cytoplasm (purple arrows). The germ nucleus outlined in bold marks the point where intercalation begins to create a single file of germ cells. (D) Schematic view of two compartments (“cells”) in the germline syncytium; note that a single plasma membrane surrounds both compartments. A germ cell has an apical pole that faces the gonad core, and an opposite or basal pole that contacts either a sheath cell as shown, or contacts the basal lamina where sheath cells are absent (see transition zone in panel A). The extracellular matrix protein HIM-4/hemicentin (red) is highly enriched along the apical faces of germ cells [115]. Note that microtubules (MTs) are oriented radially with respect to the long axis of the gonad. (E) Summary diagram of cytological changes during apoptosis, as described in this report. The full sequence occurs in engulfment-defective mutants such as *ced-1*, but sheath cell-mediated degradation in wild-type animals interrupts the late stages of the sequence at variable points. The apoptotic cell first loses mitochondria, and then shrinks while the ring channel remains open. PGL-1 (magenta) is lost rapidly from P granules, but P granules remain visible by TEM. After the ring channel closes, microtubules are remodeled into a cage-like array, and cofilin-actin rods begin to form. These changes are followed by further cell shrinkage, involving both cytoplasmic and nuclear compaction.

The anatomy and general development of the *C*. *elegans* germline has been reviewed elsewhere [8, 32–34]. In brief, the adult hermaphrodite gonad has two U-shaped, tubular arms. All images of gonads shown here depict single arms, and show longitudinal sections (Fig 1A), cross sections (Fig 1B), or tangential sections (Fig 1C). The key anatomical reference points of the gonad are the distal end, the bend or loop, and the proximal end. Germ cells divide mitotically at the distal end of the gonad, and enter successive stages of meiosis as they travel proximally. Mature oocytes form at the proximal end of the gonad, where they are ovulated into a sperm receptacle called the spermatheca (Fig 1A); germ cells in larval hermaphrodites initially differentiate as sperm (“self-sperm”) that are stored in the spermatheca, but germ cells in adults switch from spermatogenesis to oogenesis. Apoptotic cells are engulfed and removed by phagocytic sheath cells, which are large and flat somatic cells that cover most of the gonad except for a “bare zone” near the distal end (Fig 1A) [34]. In the bare zone, germ cells directly contact the basal lamina that surrounds the gonad (Fig 1D). The gonad superficially resembles a cylinder of about 1000 germ cells that surround a central, cytoplasmic region called the core (Fig 1B). More accurately, the germline is a syncytium with a single plasma membrane that forms incomplete compartments around each nucleus (Fig 1D); here, we adopt the convention of calling these compartments germ “cells”. Germ cells connect to the common core by small openings called ring channels. In the distal half of the gonad, materials move out of the germ cells and into the core [25]. In the proximal half of the gonad, materials in the core move into enlarging oogonia (Fig 1A) [25]. After reaching their full size, oogonia close their ring channels (cellularize) and become oocytes. Finally, the oocytes are fertilized either by self-sperm, or by male sperm acquired through mating. In the present study, ages of adult animals were synchronized with respect to the fourth larval stage (L4). For example a 24 hr adult is an animal that was picked as an L4 larva and analyzed 24 hrs later; a typical animal would have undergone the L4/adult molt at approximately 10 hrs.

### Apoptotic germ cells expel cytoplasm prior to closure

**Fig 2:**
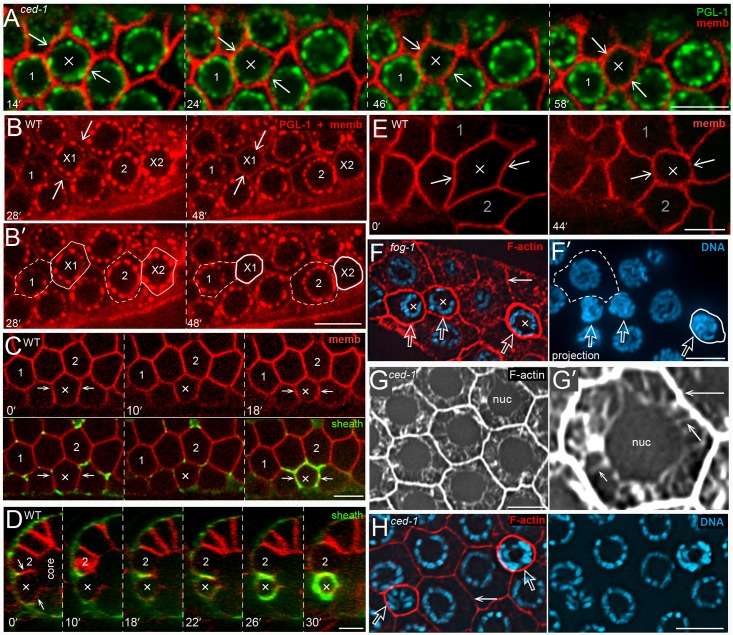
Shrinkage of apoptotic cells. (A) Video sequence of a *ced-1(e1735)* gonad expressing PGL-1::GFP (green) and a reporter for germ cell membranes (red); strains for the various transgenic reporters used here and elsewhere are listed at the ends of the respective figures legends. The images compare a non-apoptotic cell (labeled 1) with a cell (X) that underwent apoptosis during the recording. At 14 mins and at earlier timepoints the X cell is slightly larger than the non-apoptotic cell, because all germ cells are increasing in size as they move proximally (to the right). Immediately after the 14 min timepoint, PGL-1 diminishes in the X cell and the cell decreases in size. Note that two cells adjacent to, and above, the X cell also undergo apoptosis in the recording. (B) Video sequence of a wild-type gonad expressing a reporter for PGL-1 (red puncta) and a reporter for membranes (also red). Two cells (X1 and X2) underwent apoptosis during the recording, and are compared with two adjacent, non-apoptotic cells (1 and 2). The perimeters of the apoptotic cells and non-apoptotic cells are indicated by solid and dashed outlines, respectively. Both apoptotic cells begin to lose PGL-1 and decrease in size immediately after 28 mins. Note that both apoptotic cells are initially similar in size to the adjacent, non-apoptotic cells (see also S1 Fig). (C) The top row is a video sequence comparing a shrinking apoptotic cell (X) with two non-apoptotic cells (1 and 2). The bottom row shows the same cells combined with a sheath cell reporter (green). The optical plane is 1.5 microns below the outermost germ cell surface, and shows transient protrusions from the sheath cell that extend for short distances between some non-apoptotic germ cells. (D) Orthogonal view of the same gonad shown in panel C, taken through a plane that bisects the ring channel of the apoptotic cell (X). Shrinkage appears to be completed by 22 mins, but the ring channel appears to remain open until at least 26 mins. (E) Video sequence showing an enlarged oogonium (X) undergoing apoptotic shrinkage. The complete video sequence, including sheath cell engulfment, is shown in S2 Video. (F) Transformed *fog-1(q253)* mutant male gonad with oogonia, including three, shrunken apoptotic cells (X). The panel at right (F’) shows a 3 micron optical projection of the DNA channel: Engulfed apoptotic cells quickly show significant chromatin compaction, but there is relatively minor compaction in the absence of engulfment, as shown here. We use 3 micron projections where listed throughout this report to accentuate chromatin compaction. Note the high level of F-actin staining (red, phalloidin) in the shrunken apoptotic cells compared to the large, non-apoptotic cells (dashed outline). (G) F-actin staining of non-apoptotic cells. The high magnification in panel G’ indicates fine, cytoplasmic actin filaments extending between the nucleus and plasma membrane (short arrow). The intensity of staining in the cytoplasm is much less than at the cell periphery (long arrow), which is saturated in this image. (H) Comparison of F-actin staining at the perimeter of a non-apoptotic cell (solid arrow), and two apoptotic cells (open arrows) in a *ced-1(e1735)* gonad. Note that the level of F-actin is higher in the older apoptotic cell (right) than in the younger apoptotic cell (left). Bars = 5 microns. Fluorescent reporters: (A) JJ2100 + OD70, (B) JJ2212 + OD70, (C, D) OD70 + MD701.

Apoptotic germ cells in live animals usually are identified by their optical refractility as visualized by Differential Interference Contrast (DIC) microscopy, by dyes such as SYTO 12, or by the expression of transgenic reporters such as CED-1::GFP that are expressed in the sheath cells [35]. However, each of these techniques only identifies apoptotic cells that are engulfed, while a major goal of the present study was to analyze apoptotic cells that persist for long periods of time in engulfment-defective mutants. Previous studies suggested that the protein PGL-1 is a potentially useful marker for apoptosis [36]. PGL-1 is expressed only in germ cells, where it is concentrated in perinuclear granules called P granules [37]. Immunostaining experiments show that PGL-1 disappears from P granules in all apoptotic cells, although P granules remain detectable by transmission electron microscopy (TEM) until late stages of apoptosis [36, 38]. We found that PGL-1::GFP and PGL-1::RFP disappear from P granules in apoptotic cells in both engulfment-defective *ced-1* mutants and in wild-type adults (Fig 2A and 2B; S1 Video). The loss of PGL-1 occurs over an interval of about 20–30 minutes (Fig 2A), and precedes the development of optical refractility by at least 80 minutes (S1 Fig; see also [39]). We identified and analyzed apoptotic cells up to 1 hour before any detectable loss of PGL-1, but we do not know when cells first become committed to apoptosis. Prior to the loss of PGL-1, germ cells have polygonal, and usually hexagonal, shapes similar to neighboring cells, but become small and round during apoptosis (Fig 2A–2D and S1 Video). We found that the circumference of the cell begins to decrease a few minutes before, or concomitant with, the loss of PGL-1; all cells that lost PGL-1 shrank, and no cells shrank that maintained high levels of PGL-1 (Fig 2A and S1 Fig; n>100 apoptotic cells for both wild-type and *ced-1* mutant animals). Apoptotic cells varied in size before losing PGL-1, but in every case they appeared similar in size to neighboring cells (Fig 2B and S1 Fig): All germ cells gradually increase in size as they move toward the gonad loop, and then rapidly enlarge as they move through the loop and into the proximal arm of the gonad (Fig 1A). In most recordings, the apoptotic cells were located before the gonad loop and were relatively small in size. However, a few apoptotic cells had entered the loop and expanded considerably in size before losing PLG-1 and shrinking (Fig 2E and S2 Video).

Sheath cells normally contact only the basal surfaces of germ cells (Fig 1D), but sheath cells wrap around, and then fully engulf, apoptotic cells [5]. We found that wrapping was usually coincident with apoptotic shrinkage (Fig 2C and 2D), but that the extent of wrapping was highly variable; the largest apoptotic cells shrank considerably before any wrapping was evident (S2 Video). Previous studies suggested that engulfment is not necessary for shrinkage, because mutants such as *ced-1* can contain small, non-engulfed corpses [5]. In agreement, we found that the loss of PGL-1 and the rate of shrinkage in *ced-1* apoptotic cells appeared similar to that of wild-type apoptotic cells (Fig 2A and S1 Fig). We asked if sheath cells had any other roles in apoptosis by examining transformed *fog-1* male gonads; these gonads lack sheath cells, similar to wild-type males, but their germ cells differentiate as oogonia and can undergo apoptosis [5]. We found that apoptotic cells in *fog-1* male gonads appeared to lose PGL-1 and shrink similar to wild-type apoptotic cells (Fig 2F).

Previous studies showed that filamentous actin (F-actin) and non-muscle myosin are localized at the periphery of all germ cells (Fig 2G) [40] [41] [25]. In addition to peripheral localization, we found that fine actin filaments appear to extend between the germ cell nucleus and the cell periphery (Fig 2G). We could not detect the latter filaments in shrunken apoptotic cells, but there was a further increase in the level of peripheral actin [F-actin intensity for apoptotic cell/non-apoptotic cell = 1.4 +/- 0.2, n = 19; Fig 2H). Because the increase in F-actin staining occurs in both engulfment-defective *ced-1* mutants (Fig 2H) and in *fog-1* mutants that lack sheath cells (Fig 2F), the increase is within the shrinking apoptotic cells rather than the sheath cells.

**Fig 3:**
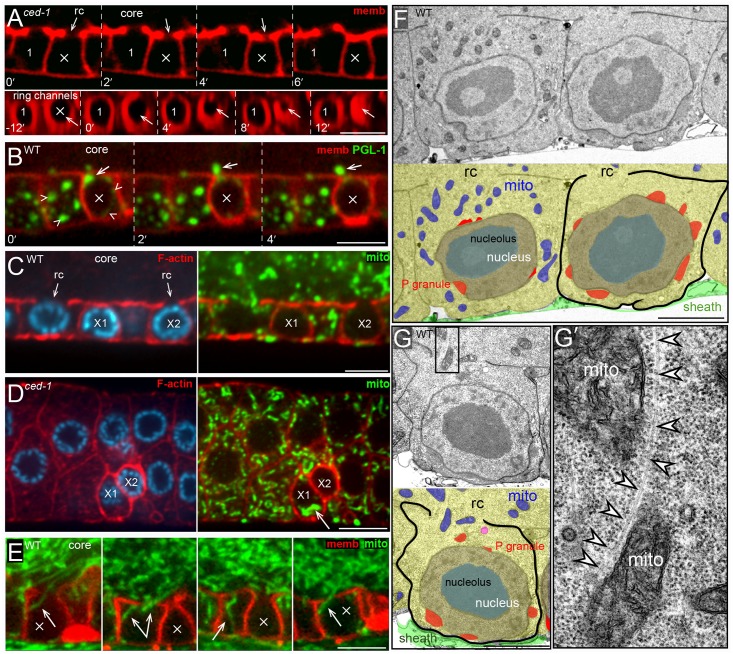
Mitochondrial exit from apoptotic cells. (A) Video sequence showing shrinkage of an apoptotic cell (X) and ring channel (rc) closure; the t = 0 timepoint here and elsewhere is the last timepoint collected before visible shrinkage. The lower row shows an orthogonal view of the same germ cells, imaged at the focal plane of the ring channels. The apoptotic cell is fully shrunken by 6 mins, but the ring channel appears to remain open until 12 mins. (B) Video sequence showing a large aggregate of cytoplasmic PGL-1 (arrow) exiting a shrinking apoptotic cell (X). The small arrowheads in the first frame mark perinuclear PGL-1 on P granules. (C) Mitochondrial ATP synthase beta (green) and F-actin (red) in a wild-type gonad. The image shows two shrunken apoptotic cells (X1 and X2) and a non-apoptotic cell (left) for comparison. X2 is younger than X1; it has less chromatin compaction, and retains an open ring channel. However, both X1 and X2 lack mitochondria. (D) Mitochondrial ATP synthase beta (green) and F-actin (red) in a *ced-1* mutant gonad. Two apoptotic cells are visible (X1 and X2); X2 lacks mitochondria similar to most apoptotic cells, but X1`contains a small clump of mitochondria (arrow). (E) Images from a live gonad expressing transgenic COX-4::GFP (green). Each panel shows a different apoptotic cell taken from the video in S3 Video. Note that the exiting mitochondria (arrows) are elongated radially with respect to the long axis of the gonad. (F) TEM micrograph of two germ cells in a wild-type gonad. Both cells appear to have similar cytoplasmic volumes and open ring channels, and neither cell is engulfed. However, the cell at right lacks mitochondria. (G) TEM micrograph of a germ cell in a wild-type gonad. This cell appears full size, but lacks mitochondria except for the two mitochondria near the ring channel. Panel G’ is a high magnification of the boxed region, and shows a microtubule (white arrowheads) next to the two mitochondria. Bars = 5 microns (A-E), 2.5 microns (F,G). Fluorescence reporters: (A) OD70 + BT24 (GFP::HIM-4, false colored red), (B) OD70 + JJ2208, (E) JJ2586.

Cell shrinkage is a hallmark of apoptosis in other systems, and is associated with nuclear/cytoplasmic compaction and convolution of the nuclear envelope [42]. Transmission electron microscopic (TEM) studies in *C*. *elegans* have shown that nuclear/cytoplasmic compaction occurs during apoptosis of somatic cells [43], and at least some apoptotic germ cells appear highly compacted [5, 44]. However, live imaging showed that germ nuclei do not decrease in size during apoptotic shrinkage (nuclear diameter before/after = 1.03 +/- .02, n = 22; S1 Fig), although different germ nuclei vary in size depending on their location in the gonad (range 3.7–5.6 microns, n = 22 apoptotic cells). Similarly, DAPI-stained apoptotic nuclei in engulfment-defective *ced-1* mutants show little if any chromatin compaction immediately after cell shrinkage (Fig 2H and see below). We used TEM to examine wild-type gonads, and identified 78 presumptive apoptotic cells that were small in size and that appeared to have closed ring channels (S2 Fig and S1 Table). One group of apoptotic cells appeared to be relatively young; these cells were only partially engulfed by sheath cells, or were engulfed and contained in phagosomes, but not phagolysosomes. A second group of apoptotic cells appeared to be older; these cells were engulfed and contained in phagolysosomes. None of the early group of apoptotic cells appeared to have cytoplasmic or nucleoplasmic compaction, and most had round nuclei (S2 Fig). Many of the older apoptotic cells were smaller than the early cells, and had convoluted nuclear envelopes and both cytoplasmic and nucleoplasmic compaction (S2 Fig). One difference between apoptosis in the gonad and apoptosis in most somatic tissues is that germ cells are part of a syncytium. We found that the ring channels in apoptotic germ cells appear to remain open during shrinkage, and for at least several minutes after shrinkage (Fig 3A). Moreover, aggregates of PGL-1 that have detached from the nuclear envelope often exit through the ring channel and into the gonad core during apoptotic shrinkage (Fig 3B; observed in about 10% of all apoptotic events). Thus, apoptotic germ cells appear to shrink in two phases: The first phase is rapid, taking only a few minutes, and results from the loss of cytoplasm through the open ring channel. The second phase of shrinkage is relatively slow, occurs after the ring channel is closed, and involves both nucleoplasmic and cytoplasmic compaction (Fig 1E).

### Selective mitochondrial transport from apoptotic germ cells

In our TEM analysis, most of the small, closed apoptotic cells lacked mitochondria (85%, S1 Table and S2 Fig), or had only one mitochondrion (14%, see panel E in S3 Fig). We also identified a few small germ cells with open ring channels that lacked mitochondria, and we presume these are shrinking apoptotic cells (S3 Fig). Consistent with these results, most of the apoptotic cells in wild-type gonads or *ced-1* mutant gonads that were immunostained for mitochondrial ATP synthase beta or Cytochrome c oxidase showed an absence of mitochondria (53% n = 84 and 75% n = 92, respectively; Fig 3C), or rarely one or a few, clumped mitochondria (Fig 3D). Unexpectedly, in both our TEM and immunostaining experiments we noticed some germ cells that similarly contained few or no mitochondria, but that showed no evidence of shrinkage and that had large, apparently normal P granules (Fig 3F and 3G). The lack of mitochondria was dependent on the apoptosis pathway, as all germ cells appear to contain numerous mitochondria in apoptosis-defective *ced-3(n717)* mutants (n = 35 gonads immunostained for ATP synthase beta). For live imaging of mitochondria, we constructed a transgenic GFP reporter for COX-4, a mitochondrial Cytochrome c oxidase subunit. Mitochondria in apoptotic cells prior to cell shrinkage or the loss of PGL-1 resemble mitochondria in non-apoptotic cells; the length of a mitochondrion can nearly equal the diameter of a cell (about 5 microns), and mitochondria often appear partially coiled around the nucleus or cell perimeter (S3 Video). As reported previously, mitochondria often undergo small shifts in position within all germ cells [25], but infrequently exit the cells (S3 Video). We found that in a typical apoptotic sequence the nucleus first shifts slightly toward the basal pole of the cell, as any intervening mitochondria shift laterally. Next, mitochondria throughout the cell body stream out of the ring channel and into the core, and are usually elongated along the direction of streaming (Fig 3E and S3 Video). Importantly, the exit of mitochondria often begins, and is sometimes complete, before any cell shrinkage is evident.

We noticed that mitochondria exiting apoptotic cells often appear to associate or fuse with mitochondria flowing in the orthogonal direction, longitudinally through the gonad core (Fig 1A and S3 Video). Thus, we wondered whether mitochondria might be pulled out of apoptotic cells through fusion with flowing, core mitochondria. Mitofusins are GTPases in the outer membrane of mitochondria that are essential for mitochondrial fusion, and *C*. *elegans* mutants lacking FZO-1/mitofusin fail to form the normal tubular networks of mitochondria in muscle cells [45]. We found that *fzo-1(tm1133)* null animals had abnormally small mitochondria in germ cells, but that these mitochondria exited apoptotic cells similar to wild-type mitochondria (0/20 *fzo-1* apoptotic cells contained mitochondria).

**Fig 4:**
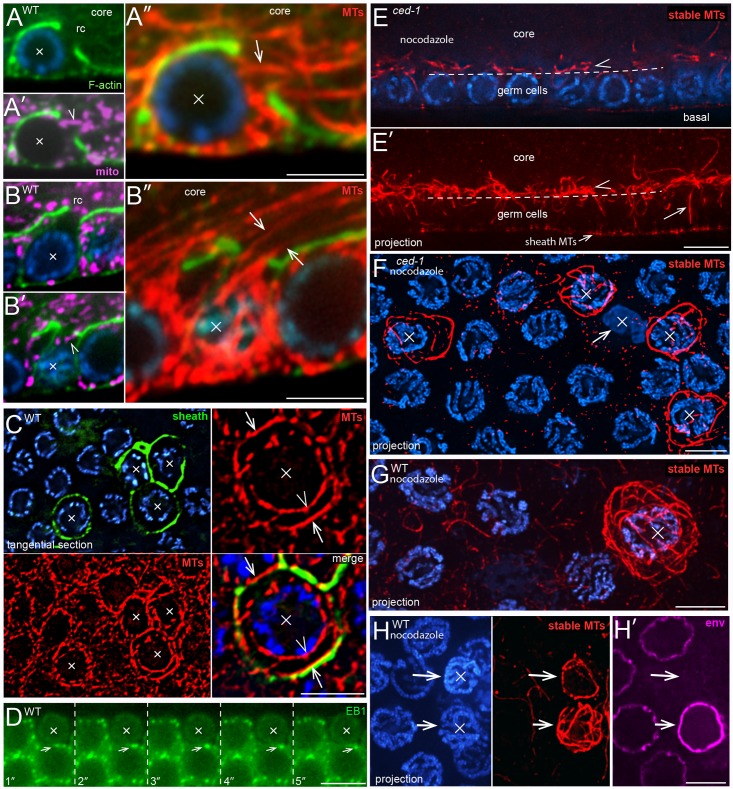
MT cytoskeleton during germ cell apoptosis. (A,B) Apoptotic cells (X) showing the MT cytoskeleton during mitochondrial exit. Both cells appear shrunken relative to neighboring cells, but both cells have open ring channels and a few mitochondria (magenta, ATP synthase beta). (A-A”) The arrowhead in panel A’ indicates a mitochondrion that appears to be exiting the apoptotic cell. Panel A” shows a high magnification of MTs (arrow) in the same focal plane. The position of this apoptotic cell is indicated in the gonad diagram in Fig 1A by the nucleus with a bold outline. Cells at this position have fewer neighbors than more distal germ cells, which simplifies the identification and tracing of single MTs associated with the cell. From 124 gonads examined, a total of three cells at this position were identified as apoptotic, and all had MTs emerging from the ring channel as shown. (B-B”) Panel B shows a focal plane through the nucleus of the apoptotic cell, and panels B’ and B” show the cortex of the same cell. (C) Wild-type gonad containing apoptotic cells (X), as indicated by their engulfment by a sheath cell reporter (green, CED-1::GFP). In the tangential optical plane shown, MTs in normal cells appear in cross section as small dots (see also Fig 1C). Note that many MTs in the apoptotic cells are parallel with the optical plane (arrows and arrowheads), and thus orthogonal to MTs in normal cells. Some of the MTs in the apoptotic cells appear closely associated with the nuclear envelope (arrowhead), while others are in the cortex (arrows). (D) Video sequence of EBP-1::GFP comets in a *ced-1(e1735)* gonad. The arrow tracks a comet in a non-apoptotic cell that is adjacent to an apoptotic cell (X). Note that the apoptotic cell has no visible comets. (E) Longitudinal optical section through a nocodazole-treated *ced-1(e1735)* mutant gonad; panel E’ shows a 5 micron projection of the same region. The dashed line indicates the boundary between the germ cells and the gonad core (compare with Fig 1A). A few, radially aligned MTs (arrow in panel E’) persist in germ cells after nocodazole treatment, but most are depolymerized. By contrast, there are numerous stable, nocodazole-resistant MTs that line the gonad core, outside of the germ cells (arrowhead). (F-H) Tangential planes of a nocodazole-treated *ced-1* gonad (panel F) and wild-type gonads (panels G and H). All MT panels show 3 micron projections, revealing most of the MTs in the cell. All of the *ced-1* apoptotic cells show a cage or cocoon-like array of stable MTs, except for a cell that appears necrotic (arrow in panel F). Engulfed wild-type apoptotic cells vary in their number of MTs (panel H): The apoptotic cell at top has compacted chromatin, few MTs, and lacks at least one nuclear epitope (magenta, NPP-9), suggesting that it has begun to degrade. Bars = 2.5 microns (A-C, F-H), 5 microns (D, E). Fluorescent reporter: (D) OD1359.

**Fig 5:**
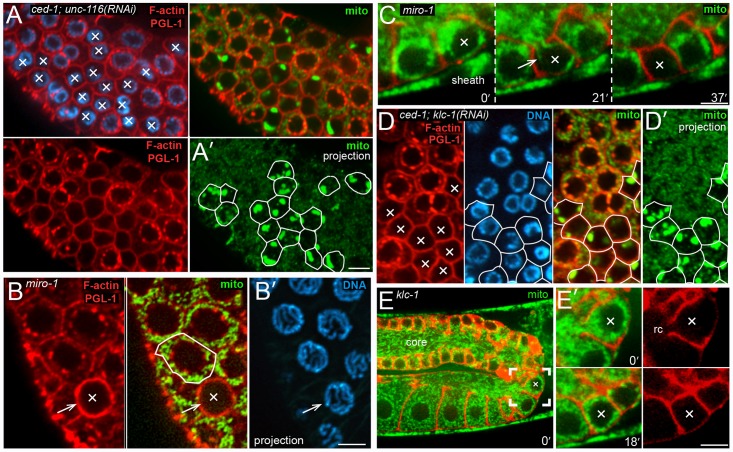
Kinesin is required for mitochondrial exit. (A) *ced-1(e1735); unc-116(RNAi)* adult gonad with a large cluster of apoptotic cells (X). The F-actin and PGL-1 signals are imaged in the same channel (red) to identify apoptotic cells by their small size and lack of PGL-1 on P granules (lower left panel). The top right panel shows mitochondria (green, ATP synthase beta) visible in the same, single optical, plane. Panel A’ shows the outlines of the apoptotic cells drawn on a 3 micron projection of the mitochondrial signal; the projection is comparable to the diameter of an apoptotic cell, and reveals most of the mitochondria within a cell. Note that the projection shows a large cluster of mitochondria in all but one of the apoptotic cells (outlined). (B) Germ cells in a *miro-1(tm1966)* null mutant, stained as for panel A. The arrow indicates the plasma membrane of an apoptotic cell (X), which lacks mitochondria. An adjacent, non-apoptotic cell is outlined for comparison. The DNA panel is a projection showing there is little or no chromatin compaction in the apoptotic cell. Thus, the apoptotic cell is at an early stage, and is unlikely to have contained, but degraded, any mitochondria. (C) Video sequence of an apoptotic cell in a *miro-1(tm1966)* null mutant expressing reporters for membranes (red) and for mitochondria (green, COX-4::GFP). Note that the exiting mitochondria (arrow) are aligned radially with respect to the gonad axis, similar to exiting mitochondria in wild-type apoptotic cells. (D) Germ cells in a *ced-1(e1735); klc-1(RNAi)* adult. The apoptotic cells (X) are outlined as for panel A. Note that all of the apoptotic cells contain clusters of mitochondria. (E) Video sequence of apoptosis in a *klc-1(ok2609)* null mutant; the complete video is provided as S4 Video. Panel E’ shows the apoptotic cell at high magnification; the ring channel is open at the first time point shown, but closes by 18 mins. Bars = 5 microns (A,D), 2.5 microns (B-C, E). Fluorescent reporter: (C, E) JJ2586 + OD70.

The linear appearance of mitochondria exiting apoptotic cells is reminiscent of the radial alignment of microtubules (MTs) in normal germ cells (Fig 1D), and we observed by TEM that mitochondria that appeared to be exiting apoptotic cells could be in close proximity to MTs (Fig 3G). We immunostained gonads for MTs and searched for cells that (1) had open ring channels, (2) contained no, or only a few, mitochondria, and (3) appeared to be at least slightly smaller in size than neighboring cells. All of these presumptive apoptotic cells appeared to have the same, radial alignment of MTs found in non-apoptotic cells (Fig 4A and 4B). In normal germ cells, MT plus ends extend through the ring channels and into the gonad core (Fig 1D). Thus, we asked whether kinesin, a plus-end-directed MT motor protein, was required for mitochondrial exit during apoptosis. *C*. *elegans* has a single, conventional kinesin heavy chain, UNC-116/KHC [46]. We found that apoptotic cells in *ced-1; unc-116(RNAi)* adults retained large numbers of mitochondria, typically aggregated into large clumps (Fig 5A; 93/94 apoptotic cells with mitochondria). Studies on kinesin-mediated traffic in neurons have shown that Miro (mitochondrial Rho GTPase) is critical for mitochondrial transport [47, 48]. Miro is associated with the mitochondrial outer membrane, where it binds the kinesin heavy chain, and the adaptor protein TRAK (trafficking kinesin-binding protein 1) [49, 50]. The *C*. *elegans* genome encodes three Miro homologs, although only MIRO-1 appears to be functional [50] [51, 52]. We found that MIRO-1/Miro did not appear to be essential for mitochondrial exit during apoptosis: Most apoptotic cells in a *miro-1(tm1966)* null mutant lacked mitochondria, similar to wild-type controls (Fig 5B; 52%, n = 91 and 53%, n = 49 respectively), or contained only a single mitochondrion. We imaged shrinking apoptotic cells in live *miro-1* mutant animals to address the possibility that mitochondria were expelled non-specifically with bulk cytoplasm, rather than by specific transport. We found that mitochondria could leave *miro-1* apoptotic cells before appreciable cell shrinkage, and that the exiting mitochondria had linear profiles similar to those in wild-type apoptotic cells (Fig 5C).

*C*. *elegans* has two kinesin light chains, KLC-1 and KLC-2, that can associate with the UNC-116/KHC-1 heavy chain; both light chains appear to mediate vesicle traffic, and KLC-1 has been shown to function in oocytes to position the meiotic spindle [53]. We found that KLC-1, but not KLC-2, appeared to be required for mitochondrial exit from apoptotic cells: Mitochondria or mitochondrial clumps were present in apoptotic cells in *ced-1*, *klc-1(RNAi)* gonads (26/26 apoptotic cells with mitochondria, Fig 5D) but not in *klc-2(RNAi)* gonads (0/17 apoptotic cells with mitochondria). Similar mitochondrial clumps were present in apoptotic cells in *unc-116(RNAi)*, *klc-1(RNAi)* gonads and in *klc-1(RNAi); klc-2(RNAi)* gonads (20–30 gonads scored for each). Live imaging of *klc-1* null mutant animals showed that few or no mitochondria exited apoptotic cells before cell shrinkage, as in wild-type gonads. Some mitochondria were lost during shrinkage, but most remained in the *klc-1* apoptotic cells when the ring channels closed (Fig 5E and S4 Video). Most of the mitochondria that were lost during shrinkage did not have the elongated, linear profiles of exiting mitochondria in wild-type apoptotic cells, suggesting that they might be expelled non-specifically with the bulk cytoplasm.

### Apoptotic germ cells reorganize and stabilize their MT cytoskeletons

Because germ cell MTs are aligned radially with respect to the gonad axes, longitudinal sections through the gonad show longitudinal profiles of MTs (Fig 1D), and tangential sections show cross-sectional profiles of MTs (Fig 1C). However, we noticed that tangential sections usually showed longitudinal profiles of MTs in apoptotic cells (Fig 4C), indicating that MT alignment changes during apoptosis. These MTs were close to the nucleus and cell cortex, forming rings inside the apoptotic cells; similar MT rings have been described for some apoptotic cells in other systems, and termed “cocoon” arrays [54]. Most MTs in *C*. *elegans* germ cells are dynamic, and the dynamics can be visualized by EB1::GFP comets (End-Binding protein 1; Fig 4D) [55] [56]. However, we found that apoptotic germ cells did not contain EB1::GFP comets (Fig 4D). The gonad also contains a population of “stable” MTs that are resistant to microtubule inhibitors such as nocodazole; most of the stable MTs line the periphery of the gonad core, and very few are found in germ cells (Fig 4E) [29, 57]. By contrast, we found that most of the MTs in apoptotic cells were resistant to nocodazole (Fig 4F–4H). Nearly all of the apoptotic cells in *ced-1* mutants contained stable MTs, except for a few cells that appeared necrotic (Fig 4F). However, several apoptotic cells in wild-type gonads contained few, or no, stable MTs (Fig 4H). These results suggest that apoptotic germ cells begin to reorganize and stabilize their MT cytoskeleton after cell shrinkage, but that this process is aborted in wild-type gonads by engulfment and degradation. Microtubule arrays in mitotic cells and in several non-mitotic cell types are nucleated by centrosomes (centrioles plus pericentriolar material), although MTs in the postmitotic germ cells of *C*. *elegans* appear to be nucleated at non-centrosomal sites at the plasma membrane and nuclear envelope [57, 58]. In our TEM study, we found 19 non-apoptotic cells where one or both centrioles were visible in the plane of section; as expected, there were no MTs adjacent to the centrioles in any of these cells (S4 Fig). Interestingly, four of nine apoptotic cells with visible centrioles had a least one MT adjacent to a centriole (S4 Fig; see Discussion).

### Apoptotic cells reorganize and stabilize actin into giant, cofilin-actin rods

**Fig 6:**
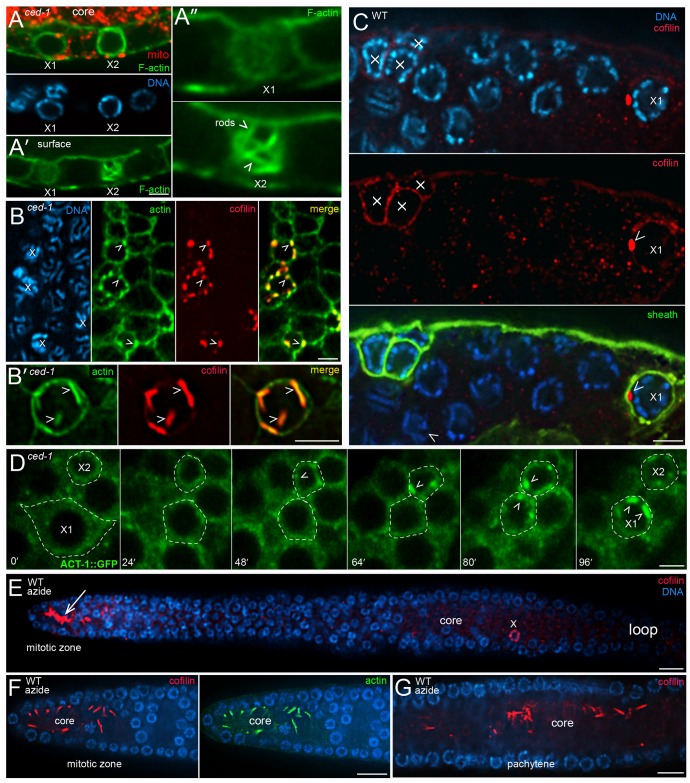
Cofilin-actin rods in apoptotic germ cells. (A) *ced-1(e1735)* mutant gonad stained for F-actin (green, phalloidin) and immunostained for mitochondria (red, ATP synthase beta). The image shows two apoptotic cells (X1 and X2) that are fully shrunken, closed, and lack mitochondria. X1 has little or no chromatin compaction, and thus appears younger than X2. However, the level of F-actin staining at the periphery of X2 is higher than X1, suggesting that the level of actin continues to increase after cell shrinkage and closure. Panel A’ shows an optical plane taken at the surfaces of the cells, and panel A” shows higher magnifications of these surfaces. Note that the actin in the older apoptotic cell, X2, appears concentrated in multiple, rod-like structures. (B) *ced-1* mutant gonads immunostained for actin (green, anti-actin) and cofilin (red, anti-UNC-60A); examples of cofilin-actin rods are indicated by arrowheads. Panel B’ is a higher magnification, surface view of a single apoptotic cell. (C) Wild-type gonad immunostained for cofilin (red, anti-UNC-60/cofilin) and sheath-specific GFP (green, CED-1::GFP). Four apoptotic cells are visible, as indicated by their engulfment by a sheath cell. The apoptotic cell labeled X1 has a single, prominent cofilin-actin rod (arrowhead). The apoptotic cells at left lack obvious rods, but appear to have higher levels of cytoplasmic cofilin than neighboring, non-apoptotic cells. (D) Video sequence of a live, *ced-1(e1735)* gonad expressing a transgenic reporter for actin (ACT-1::GFP). Two apoptotic cells (X1 and X2) are visible; X2 has completed shrinkage at the first timepoint, and X1 completes shrinkage at about 48 mins. Cofilin-actin rods (arrowheads) begin to form in X2 at about 48 mins, and in X1 at about 80 mins; X1 shifted below the focal plane at t = 96 mins. (E) Azide-treated, wild-type gonad, immunostained for cofilin (red, anti-UNC-60A). The apoptotic cell (X) contains rods, as in untreated gonads, but azide has induced rod formation in the gonad core in the mitotic region. The lower panels show confocal images of rods induced in mitotic region (panel F) and the pachytene region (panel G). Note that the induced rods form in the gonad core, and not within germ cells. Rod induction in the mitotic region was observed in the following gonads after exposures to 20 mM and 50 mM azide: 2 hrs azide (0/12, 2/32 gonads), 3 hrs (3/16, 30/36), 4 hrs (2/9, 1/35), 5 hrs (4/9, 7/31), 6 hrs (1/15, 1/30), 7 hrs (4/9, 2/25), 8 hrs (0/24, 0/24). Rod induction was observed in the pachytene region after a 3 hr treatment with azide (1/16, 2/36). No cofilin-actin rods were induced in gonads treated with 10 mM azide for 30 mins, 1 hr, and 2 hrs (n = 12–22 gonads, each). About 30% of animals treated for 8 hrs with 50 mM azide recovered some locomotion after removal from azide. Bars = 2.5 microns (A-D), 5 microns (E). Fluorescent reporters: (C) MD701, (D) JJ1477.

**Fig 7:**
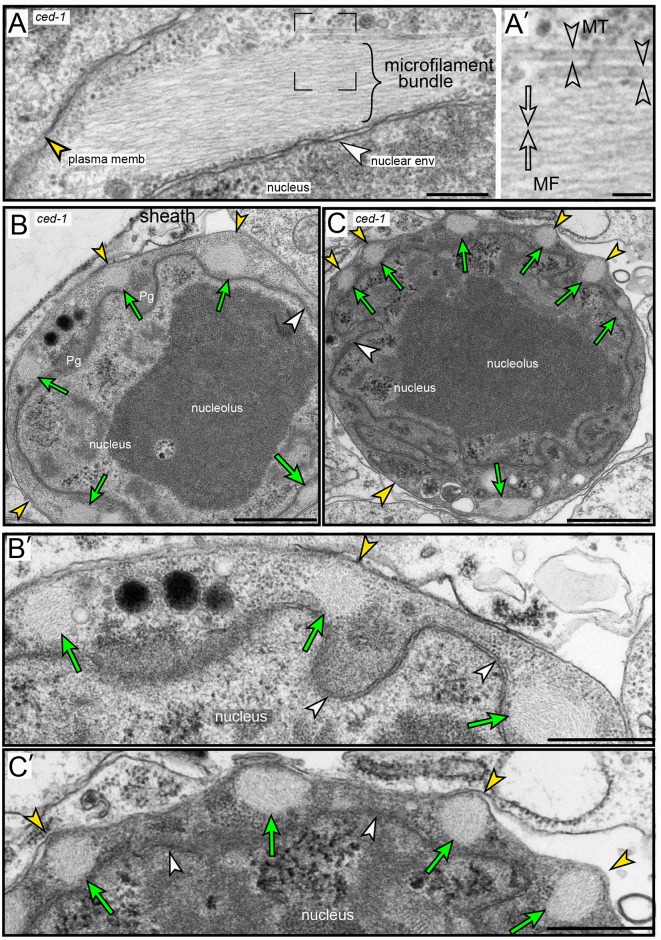
Microfilament bundles in *ced-1* apoptotic cells. (A-C) TEM micrographs of *ced-1(e1735)* apoptotic cells showing microfilament bundles. The plasma membrane (yellow arrowhead) and the nuclear envelope (white arrowhead) of the apoptotic cell are indicated in all panels. (A) Example of a single bundle; a higher magnification of the boxed region is shown in panel A’, and indicates a single microfilament (open arrows) and two MTs (open arrowheads). (B and B’) Apoptotic cell with multiple microfilament bundles (green arrows), visible as cross sections; note that the surface of the cell is relatively smooth. (C and C’) Older apoptotic cell, as indicated by the convoluted nuclear membrane and increased cytoplasmic density. Note that the surface has bumps or ridges overlying each of the microfilament bundles. Bar = 200 nm (A), 50 nm (A’), 1 micron (B,C); 0.5 micron (B’, C’).

We found that apoptotic germ cells also remodel their actin cytoskeleton. As described above, the level of phalloidin-stained F-actin increases at the periphery of *ced-1* apoptotic cells during shrinkage (Fig 2H). Surprisingly, the level of phalloidin staining appeared to increase further after shrinkage and closure in a few *ced-1* apoptotic cells. For example, some *ced-1* apoptotic cells with compacted chromatin showed much higher levels of phalloidin staining than presumably younger apoptotic cells that were fully shrunken but had little or no chromatin compaction (Fig 6A). The increase in phalloidin staining was highly variable, and occasionally was concentrated in multiple, rod-like structures (Fig 6A). To resolve this variability, we processed *ced-1* mutant gonads for TEM using a conventional glutaraldehyde/osmium fixation protocol. Among 131 *ced-1* apoptotic cells analyzed, about 70% had one or more giant bundles of microfilaments that were not observed in any non-apoptotic cells (Fig 7A–7C, S5 Fig, and S1 Table). Most of the *ced-1* apoptotic cells that lacked microfilament bundles appeared to be young cells with round nuclei, or were advanced cells with necrotic features such as a perforated nuclear envelop (S1 Table). Individual microfilaments within a bundle were 6–10 nm in thickness, consistent with the dimensions of filamentous actin, and the larger bundles were 200–300 nm in diameter (Fig 7A and S5 Fig). The microfilament bundles often appeared to terminate at the plasma membrane or the nuclear envelope, and several of the bundles extended directly between the plasma membrane and the nuclear envelope (S5 Fig). The smallest apoptotic cells with cytoplasmic and nuclear compaction often had ridges or bumps on the cell surface, overlying the microfilaments bundles (compare Fig 7B with Fig 7C).

Giant microfilament bundles that show variable, or no, staining with phalloidin have been described in mammalian cells, where they are termed cofilin-actin rods [59]. Cofilin/ADF (Actin-Depolymerizing Factor) proteins are best known for their actin-severing activity. However, cofilin that is dephosphorylated at a conserved, N-terminal Ser3 residue can instead promote actin bundling; the dephosphorylated cofilin alters the actin filament conformation and inhibits binding of phalloidin [60]. We immunostained *ced-1* gonads with an antibody specific for the C-terminus of *C*. *elegans* UNC-60A/cofilin [61], and used an anti-actin antibody, rather than phalloidin, to visualize actin. Most of the actin in the apoptotic cells was localized in large, rod-like bodies (S5 Video) that co-localized with cofilin (Fig 6B); we term these structures cofilin-actin rods. Similar cofilin-actin rods were observed in most apoptotic cells in the following engulfment-defective mutants: *ced-2(1752)*, *ced-5(n1812)*, and *ced-7(n1892)* (n = 42–68 gonads, each). Conversely, cofilin-actin rods were not detected in any germ cells in apoptotic-defective *ced-3(n717)*, *ced-3(n3692)*, *or ced-4(n1162)* mutants (n = 32–45 gonads). Apoptotic cells in wild-type adults contained few or no cofilin-actin rods, but often had a level of cytoplasmic cofilin/UNC-60A staining that appeared higher than in neighboring, non-apoptotic germ cells (Fig 6C). We did not notice microfilament bundles in our initial TEM examination of wild-type apoptotic cells. In a second survey at higher magnification, however, we found that about 10% of wild-type apoptotic cells contained one or a few, small bundles of microfilaments (S5 Fig, 6/65 apoptotic cells). To determine the dynamics of cofilin-actin rod formation, we imaged live *ced-1* mutant adults containing a transgenic reporter for actin (Fig 6D). This analysis showed that rods become visible in apoptotic cells about 40–70 minutes after shrinkage and closure (n = 8 apoptotic cells; Fig 6D). Together, these results suggest that (1) apoptotic cells begin remodeling their actin cytoskeleton into cofilin-actin rods shortly after shrinkage and closure, (2) apoptotic cells that undergo cytoplasmic and nucleoplasmic compaction after closure develop surface bumps/ridges above the rods, and (3) that the engulfment and degradation of apoptotic cells in wild-type gonads aborts further development of the rods.

Cofilin-actin rods in humans are observed in myopathies and in neural pathologies such as Alzheimer’s and Huntington’s disease [62], and can be induced experimentally in some types of cultured cells by stresses that include ATP depletion [63]. Because apoptotic germ cells lose mitochondria, the principal source of ATP, we wondered whether ATP depletion alone would trigger rod formation in otherwise normal germ cells. Azide is a potent inhibitor of mitochondrial respiration in *C*. *elegans*; for example, a 30 min exposure to 10 to 40 mM sodium azide decreases ATP levels by about 50% to 80%, respectively [64]; although each of these azide concentrations paralyzes *C*. *elegans*, animals appear to recover fully after the azide is removed. We immunostained gonads for actin and cofilin after treating wild-type adults with varying concentrations of 10–50 mM azide for 30 mins to 8 hrs. We found that azide did not cause a general induction of cofilin-actin rods within germ cells in the pachytene region, where cells normally undergo apoptosis (Fig 6E). However, azide often induced cofilin-actin rods in the core of the mitotic zone (Fig 6E and 6F), and rarely in the core of the pachytene region (Fig 6G, quantitation in Figure legend). These results suggest that rod formation in apoptotic cells requires additional events beyond ATP depletion.

### Cofilin-actin rods identify a novel population of binucleate apoptotic germ cells

**Fig 8:**
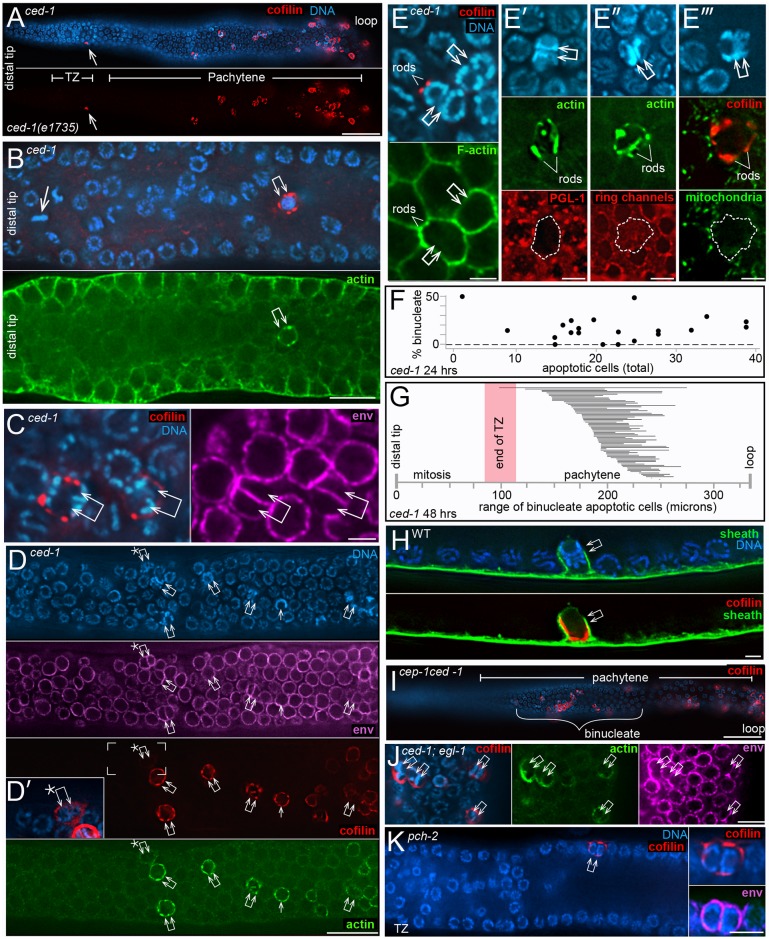
Binucleate apoptotic germ cells. (A) *ced-1(e1735)* mutant gonad showing rod-containing cells throughout the mid-pachytene region, and one rod-containing cell near the border between the transition zone and the early pachytene region (arrow). (B) High magnification of the distal region of a *ced-1* mutant gonad. The double arrow points to a binucleate, rod-containing cell in the transition zone. Note that this cell is only about 12 cell diameters away from a mitotic cell at metaphase (single arrow). Similar distal, rod-containing cells were found in the transition zones in 5/178 gonads. (C) Two examples of rod-containing cells (double arrows) in the mid-pachytene region of the gonad. Note that both cells have two nuclei, as indicated by the chromatin patterns and nuclear envelopes (magenta, anti-NPP-9). (D) Mid-pachytene region of a *ced-1* gonad showing multiple binucleate cells (double arrows). Cofilin-actin rods are visible in all of the binucleate cells except for the distalmost cell (asterisk), which has a higher level of cytoplasmic cofilin than in neighboring, non-apoptotic cells (inset in Panel D’). Note that the two nuclei in many of the binucleate cells are aligned in the same, surface plane as other gonad nuclei. As described below, this alignment contrasts with the radial alignment of the nuclei when binucleate cells first form. (E-E”‘) Each combination of panels depicts apoptotic-specific features of the binucleate, rod-containing cells (double arrows); the cofilin-actin rods are indicated by either UNC-60/cofilin (red in E and E”‘) or by actin (green in E’ and E”). The rod-containing cells are small with high levels of peripheral F-actin (panel E), they lack PGL-1 on P granules (panel E’), they have closed ring channels (panel E”), and they lack mitochondria (panel E”‘). (F) Graph showing the percentage of binucleate, rod-containing apoptotic cells compared to the total number of apoptotic cells in *ced-1* gonads. The data is from single gonad arms in 21 *ced-1* adults at 24 hrs. (G) Diagram showing the range of positions of rod-containing, binucleate, apoptotic cells in *ced-1* mutants at 48 hrs. Each horizontal bar represents one of 73 gonad arms analyzed; the left endpoint indicates the position of the distalmost, binucleate apoptotic cell, measured from the distal tip of the gonad. The right endpoint is the position of the proximal-most apoptotic cell that could be scored as binucleate; other apoptotic cells might also have been binucleate, but their chromatin was too compacted to score. In the set of gonads analyzed, the proximal boundaries of the transition zones (TZ) ranged from 80–115 microns, and the gonad loops were at 336 +/- 23 microns. (H) Engulfment of a binucleate, rod-containing apoptotic cell in the mid-pachytene region of a wild-type gonad. The gonad is from an MD701strain expressing the sheath reporter CED-1::GFP, and is here immunostained for GFP (green). (I-K) Binucleate, apoptotic cells in mutants that have physiological apoptosis, but lack other forms of apoptosis. Binucleate, apoptotic cells were present in the following 48 hr adults: *cep-1(gk138*) (7/21 gonads), *cep-1(gk138) ced-1(RNAi)* (26/26 gonads, panel I), *ced-1(e1735); egl-1(n1084n3082)* (32/32 gonads, panel J), *egl-1(n1084n3082)* (7/28 gonads), and *pch-2(tm1458)* (8/26 gonads, panel K). Bars = 20 microns (A, D, I), 10 microns (B), 2.5 microns (C, E, H), 5 microns (J-K).

Physiological apoptosis in *C*. *elegans* is widely reported as occurring only at or after the late-pachytene stage of meiosis, near the loop region of the gonad [65] [32] [66] [67] [9] [68]. Indeed, for this reason we did all of our live imaging studies near the gonad loop. Most of the cells with cofilin-actin rods in *ced-1* gonads were near the gonad loop, as expected (Fig 8A). However, other rod-containing cells were in the mid-pachytene region, and occasionally in the early pachytene region (Fig 8A and 8B). Remarkably, all of the rod-containing, mid-pachytene cells appeared to be binucleate, or rarely trinucleate (Fig 8C and 8D and S6 Video). Because our above results show that cofilin-actin rods do not occur in apoptosis-defective *ced-3* mutants, we presumed that the rod-containing binucleate cells were apoptotic. Indeed, these cells have the following apoptosis-specific features: (1) compacted chromatin, (2) lack of PGL-1 on P granules, (3) relatively small cell size with little cytoplasm, (4) an F-actin-rich periphery, (5) a closed ring channel, and (6) no mitochondria (Fig 8E). Binucleate apoptotic cells were found in 85% of *ced-1* gonads at 24 hrs (n = 20 gonads) and 100% at 48 hrs (N = 73 gonads). Comparable numbers of binucleate apoptotic cells were observed in 48 hr adults in each of the following engulfment-defective mutants: *ced-2(1752)*, *ced-5(n1812)*, and *ced-7(n1892)* mutants (n = 52–64 gonads, each). We found that *ced-1* adults of a given age varied in the percentage of total apoptotic cells that were binucleate (Fig 8F), and varied in the spatial distribution of binucleate apoptotic cells (Fig 8G). Binucleate apoptotic cells also were present in wild-type gonads, where they were engulfed and removed by sheath cells (Fig 8H). Finally, we searched for, and identified, binucleate cells at various stages of apoptosis in our TEM preparations of both wild-type gonads and *ced-1* gonads (S6 Fig and S1 Table).

**Fig 9:**
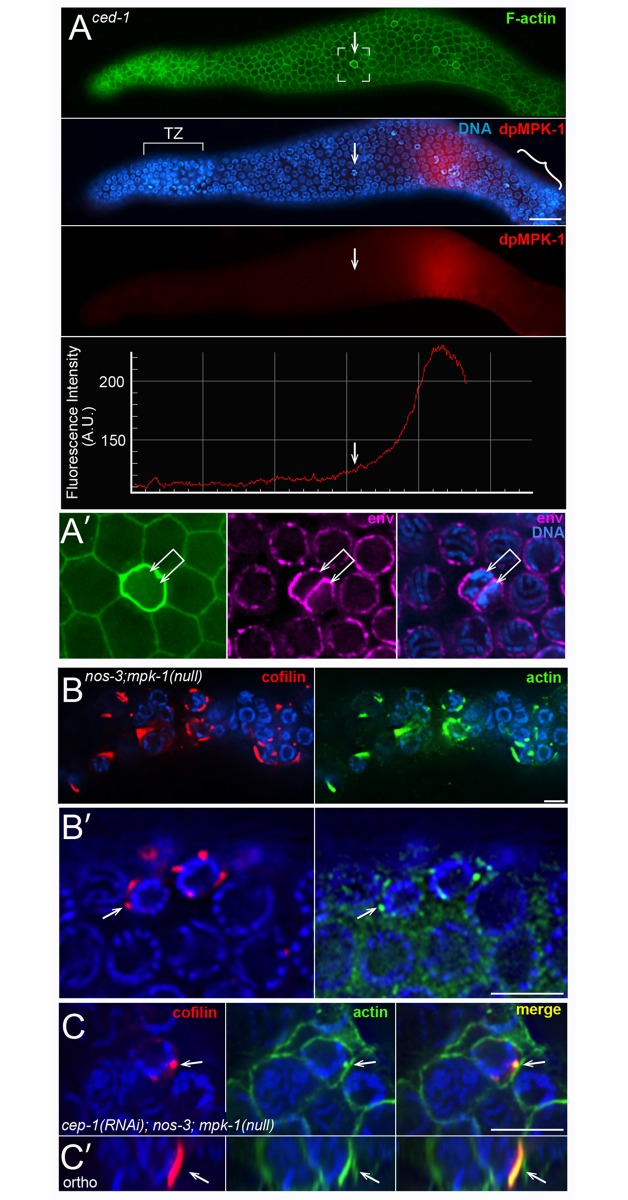
Binucleate apoptotic cells and MAP kinase. (A) *ced-1(e1735)* gonad at 48 hrs immunostained for activated MAP kinase (red, anti-dpMPK-1) and F-actin (green, phalloidin). The boxed region contains a binucleate apoptotic cell (arrow), as shown at higher magnification in panel A’ (the nuclear envelope is shown in magenta, anti-NPP-9). Note that this cell is located before the peak of activated MAP kinase, although most apoptotic cells (bracketed region in panel A) occur after the peak. Additional examples of binucleate apoptotic cells and dpMPK-1 are presented in S7 Fig. (B and C) Cell deaths in a *nos-3(oz231); mpk-1(ga117)* null gonad (panel B), and in a *cep-1(RNAi); nos-3(oz231); mpk-1(ga117)* gonad (panel C). The presumptive apoptotic cells are small, have closed ring channels, and have compacted chromatin. Panel C’ shows an orthogonal view of the rod indicated in panel C (arrow). Bars = 20 microns (A), 5 microns (A’, B-C).

Physiological apoptosis is genetically distinct from apoptosis caused by stresses such as DNA damage or synaptic failure, and is distinct from the apoptosis of somatic cells. These other forms of apoptosis require CEP-1 (a *C*. *e**legans* p53 homolog), PCH-2 (a homolog of human TRIP13), or EGL-1 (a BH3-only apoptosis effector) [69–71]. We found binucleate apoptotic cells in *cep-1* mutants, in *egl-1* mutants, and in *pch-*2 mutants (Fig 8I–8K), suggesting that physiological apoptosis includes the removal of binucleate germ cells. Studies on strong, loss-of-function *mpk-1*/Map Kinase mutants have suggested that physiological apoptosis requires a MAP-kinase activation pathway [5], although other studies with a temperature-sensitive *mpk-1* allele suggested that MPK-1 might inhibit, or both promote and inhibit, apoptosis [72]. Wild-type gonads that are immunostained with an antibody specific for the activated, diphosphorylated form of MPK-1/Map Kinase (hereafter dpMPK-1) show a strong peak of staining in the late-pachytene region [27] [26] [73], and we observed a similar staining pattern in *ced-1* mutant gonads (Fig 9A and S7 Fig). The majority of apoptotic cells occur after the peak expression of dpMPK-1, near the gonad loop (Fig 9A and S7 Fig). However, most of the binucleate apoptotic cells are located before the peak of dpMPK-1 (Fig 9A and S7 Fig). *mpk-1* mutants have multiple germline defects that potentially complicate an analysis of apoptosis; for example, *mpk-1* mutants have severe defects in membrane organization [73]. Recent studies have shown that mutations in the *nos-3* gene are capable of partially suppressing these membrane defects [74]. We examined *nos-3;mpk-1(null)* and *cep-1(RNAi); nos-3; mpk-1(null)* gonads, and found several examples of presumptive apoptotic cells; these cells had cofilin-actin rods, were smaller than neighboring cells, had closed ring channels, and had compacted chromatin (Fig 9B and 9C). We anticipated that the apoptotic cells would be binucleate, but nearly all had a single nucleus. Thus, these results support a hypothesis that MPK-1 has an indirect role in some deaths that occur by physiological apoptosis, possibly by maintaining membrane integrity.

### Fate of binucleate cells in apoptosis-defective mutants

**Fig 10:**
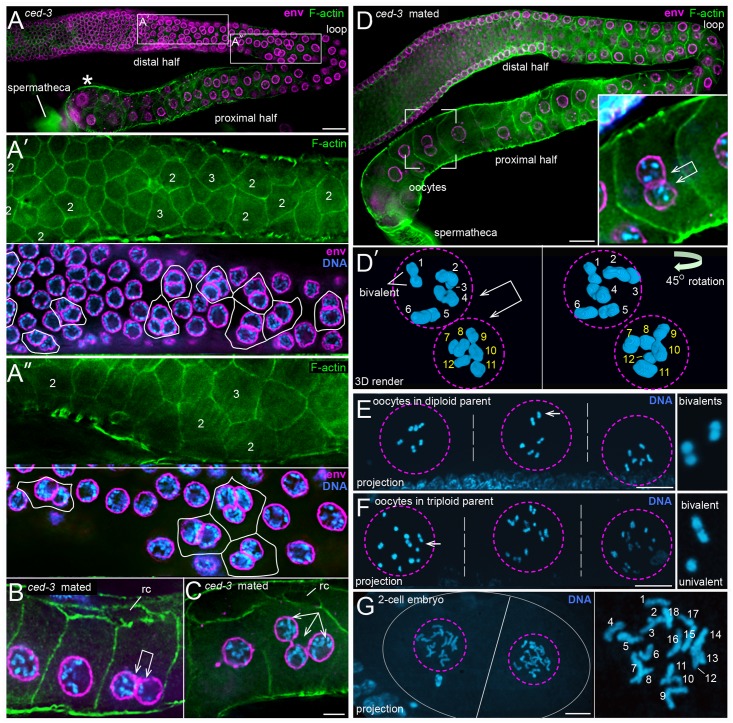
Non-apoptotic, binucleate germ cells in *ced-3* mutant gonads. (A-A”) Gonad from *ced-3(n3692)* adult at 72 hrs stained for F-actin (green, phalloidin) and for the nuclear envelope (magenta, anti-NPP-9). The proximal half of the gonad contain numerous, abnormally small oocytes, but does not contain any binucleate oocytes. The two boxed regions from the distal half of the gonad are shown in panels A’ and A” at higher magnification. The numbers indicate binucleate (2) or trinucleate (3) germ cells, as inferred from the nuclear channel. (B and C) Gonads from mated *ced-3(n3692)* adults at 72 hrs, showing a binucleate oogonium (panel B) and a trinucleate oogonium (panel C). Both oogonia are enlarged, but are not full-size oocytes; note open ring channels (rc). (D) Gonad from a mated *ced-3(n3692)* adult at 72 hrs. Oogonia in the proximal half of the gonad have better organization than in the gonad in panel A, but they do not form the normal, single file. A full-size, binucleate oocyte is visible in the boxed region, and shown at higher magnification in the inset. Panel D’ is a 3D rendered view of an optical stack through the same binucleate oocyte. The second panel is rotated 45 degrees to show all six bivalents in each nucleus. (E and F) Adult progeny from a cross between a wild-type male and a *ced-3(n3692)* hermaphrodite. Each image shows three adjacent oocytes (separated by dashed white lines) in a gonad arm; each oocyte contains a single nucleus (outlined by a dashed magenta circle). The adult in panel E is diploid, as each oocyte contains six bivalent chromosomes; the panel at right shows a higher magnification of two bivalents. The adult in panel F is a triploid, as each oocyte contains six bivalents plus six univalents; the panel at right shows a higher magnification of one bivalent and one univalent. The low magnification images are 5 micron projections, such that all chromosomes are visible. (G) 2-cell embryo in the uterus of a triploid adult as in panel F. Each nucleus in the 2-cell embryo contains 18 chromosomes, as expected for the self-progeny of a triploid hermaphrodite. The image is a projection of an optical stack through the nuclei. Bars = 10 microns (A, B), 5 microns (C-F).

We wondered whether pachytene-stage binucleate germ cells that undergo apoptosis in wild-type gonads would instead progress to become binucleate oocytes in apoptosis-defective mutants. Pachytene-stage binucleate germ cells were observed in 100% of 48 hr *ced-3(3692)* and *ced-4(n1162)* gonads (n = 28 and 23 gonads, respectively; Fig 10A). These gonads also contained several trinucleate germ cells, which are rare in wild-type gonads (Fig 10A). However, most of the *ced-3* and *ced-4* gonads did not contain any binucleate or trinucleate cells in the proximal half of the gonad, where oogonia enlarge and cellularize to become oocytes. Indeed, we did not find any binucleate oocytes in unmated *ced-3(n3692)* adults (n = 2771 oocytes), or in control wild-type adults (n>4000 oocytes). Because many of the 72 hr *ced-3* adults appeared to have ceased ovulation, as occurs when hermaphrodites deplete their store of self-sperm, we examined mated adults. These gonads contained a few enlarged binucleate and trinucleate oogonia (Fig 10B and 10C), and rare binucleate oocytes (Fig 10D; n = 3 binucleate oocytes in 72 gonads). Wild-type oocyte nuclei have 6 pairs of homologous chromosomes (bivalents) at the diakinesis stage of prophase [75], and we found that each of the two nuclei in the binucleate oocytes had 6, diakinetic bivalents (Fig 10D).

We next wanted to determine if the binucleate *ced-3* oocytes could produce viable offspring when fertilized by wild-type sperm; wild-type males were chosen for the mating to prevent the occasional larval lethality reported for *ced-3* homozygous animals [76]. We anticipated that a binucleate oocyte that was fertilized by a normal sperm might become a triploid animal: Wild-type hermaphrodites are diploid, with two X chromosomes and two sets of autosomes (2X; 2A), and wild-type males have a single X chromosome (1X:2A). Triploids have been produced experimentally in *C*. *elegans* by crossing 4X;4A tetraploid hermaphrodites with 1X;2A wild-type males [77]. The resulting triploid progeny are viable, and become fertile hermaphrodites (3X;3A) when the oocyte is fertilized by an X-bearing sperm, or become males (2X;3A) when the oocyte is fertilized by a nullo-X sperm. Because binucleate *ced-3* oocytes are rare, we set up multiple mating pools of *ced-3(n3692)* hermaphrodites crossed with wild-type males. The resulting progeny were grown to adulthood, then fixed and stained with DAPI to determine ploidy. We limited our analysis to hermaphrodite progeny, and scored adult ploidy by the number of chromosomes in oocyte nuclei; oocytes from diploid parents have 6 bivalents, and oocytes from triploid parents are expected to have 6 bivalents plus 6 univalents [77]. The vast majority of adult hermaphrodite progeny from the *ced-3* cross were diploid (99.7%, n = 1360 adult progeny), as were all of the wild-type progeny from a control cross (100%, n = 4869 adult progeny) (Fig 10E). However, four adult progeny from the *ced-3* cross were triploid (Fig 10F). Each of the triploid adults appeared morphologically normal and contained developing embryos, including one 2-cell embryo at mitotic prophase (Fig 10G). Both cells in the 2-cell embryo contained 18 chromosomes, as expected for a triploid embryo, rather than the normal, diploid number of 12 chromosomes (Fig 10G).

### Origin of binucleate germ cells

**Fig 11:**
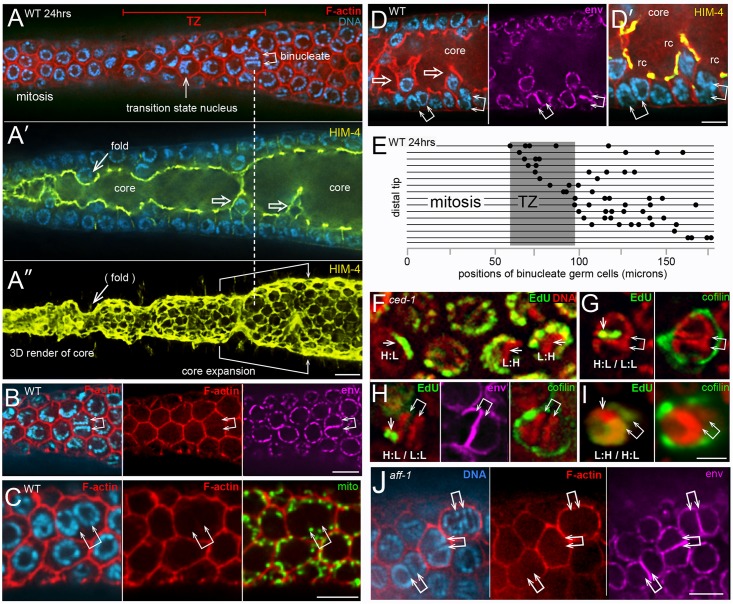
Origin of binucleate cells. (A-A”) Distal end of wild-type gonad stained for F-actin (red), HIM-4 (yellow), and DNA (blue). The chromatin in an interphase mitotic nucleus forms a symmetrical sphere around the large, central nucleolus. Chromatin in a transition state (TZ) nucleus has an asymmetric, crescent shape, caused by chromosomes pairing. Panel A shows a binucleate germ cell (double arrow) next to the transition zone; note that both nuclei in the binucleate cell resemble transition state nuclei. Panel A’ shows an optical section through the middle of the same gonad; note the enrichment of HIM-4 at the periphery of the gonad core (see also diagram in Fig 1D). Panel A” is a 3D rendered view of an optical stack through the gonad core; the ring channels appear as small ovals on the surface of the core. Note that the ring channel of the binucleate cell (dashed vertical line) is larger than in surrounding germ cells. Although most germ cells contact the periphery of the gonad, several appear folded into the gonad core (open arrows in panel A’). The folding pattern can be inferred from the 3D rendered view of the core, where grooves or depressions in the core correspond to folds. Note that the gonad core expands significantly as the folds disappear. (B-D) Each row of panels shows distal binucleate cells lacking features of apoptotic cells as follows: They have normal levels of peripheral F-actin (panel B), they contain mitochondria (panel C), and they have open ring channels (panel D). Note in panel D that both binucleate cells (double arrows) are at the bases of small folds (open arrows). (E) Map showing the positions of binucleate, non-apoptotic cells (dots) in the distal gonad; each horizontal bar represents one gonad. Importantly, binucleate cells could be distinguished easily from mitotic germ cells in anaphase or telophase (S8 Fig). (F-I) Each set of panels shows EdU (green) that was incorporated into replicated DNA (red). Panel F shows a *ced-1(e1735)* gonad fixed and stained immediately after 45 mins of labeling. Germ nuclei show H:L or L:H patterns as indicated and described in the text; paired X chromosomes are indicated by an arrow. Panels G-I show binucleate apoptotic cells (indicated by cofilin-actin rods) 20 hours after a pulse of EdU. Note that the two nuclei in a binucleate cell have different labeling patterns, as indicated. (J) Binucleate germ cells in an *aff-1(tm2214)* mutant gonad. Bars = 5 microns (A-E), 2.5 microns (F-I).

We found that the binucleate apoptotic cells in wild-type adults and in *ced-1* adults originate from binucleate, non-apoptotic cells in the distal third of the gonad (Fig 11A). The non-apoptotic, binucleate cells resemble other non-apoptotic cells in that they (1) contain numerous mitochondria, (2) have a single, open ring channel, and (3) have PGL-1 on P granules (Fig 11B–11D). Conversely, they do not have apoptotic-specific features such as cofilin-actin rods, compacted chromatin, or an enrichment of peripheral F-actin (Fig 11B–11D; 25–40 gonads scored for each feature). However, the binucleate cells are slightly larger than neighboring cells (Fig 11C), they often have a larger than normal ring channel (Fig 11A and 11D), and they contain two pairs of centrioles rather than one pair (S4 Fig). We hypothesized that the distalmost binucleate cells might be located near their origin. Accordingly, we mapped the positions of all binucleate, non-apoptotic cells within a 180 micron region from the distal end of the gonad; this region contains the mitotic zone and the transition zone, where germ cells enter meiosis (Fig 11E). We did not find binucleate cells in the mitotic zone. Instead, the distalmost binucleate cells were either in, or on the proximal side of, the transition zone (Fig 11E). We attempted to determine if the two nuclei in a binucleate cell were sister nuclei by pulse labeling replicating DNA with the thymidine analog 5-Ethynyl-2’-deoxyuridine (EdU); our scoring protocol should preferentially identify cells that were labeled in mitotic S phase rather than meiotic S phase (see Methods). Because X chromosomes replicate at a different time than autosomes [6], most normal germ cell nuclei have a pattern of EdU labeling that is either L:H (Low X: High autosomes) or H:L (High X: Low autosomes) depending on the time of labeling relative to the cell cycle (Fig 11F). If a binucleate cell originates from a single, labeled parent, we expected that the two nuclei should both be labeled, and have the same pattern of labeling. Instead, most of the binucleate cells contained only one labeled nucleus (15/17 binucleate cells, Fig 11G and 11H). Of the minority of binucleate cells with two labeled nuclei, the patterns of labeling were different (Fig 11I). Together, these results suggest that the binucleate cells do not arise from incomplete cell division, but rather by the fusion of postmitotic germ cells.

**Fig 12:**
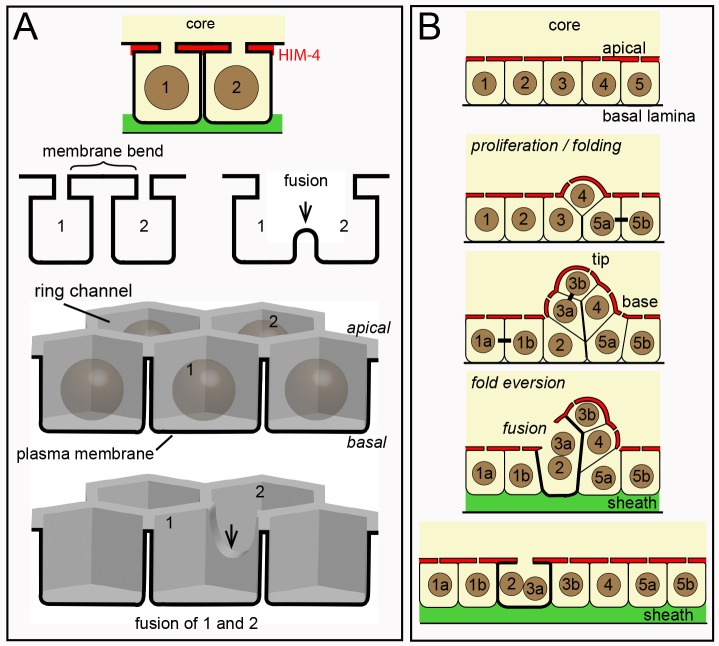
Interpretative diagram of folding, eversion, and fusion in the germline syncytium. (A) Diagram representing possible membrane topology during a fusion event. Germ “cells” are nuclear compartments divided by saddle-like bends in a single plasma membrane (bold line). A reduction of the saddle opens the compartments between cells 1 and 2. The lower diagram shows a perspective view of the same fusion. (B) Cell division (bars between cells) and growth causes the germline syncytium to fold or buckle inward. Many or most mitotic spindles are not aligned radially, so the folds do not appear to be created by directional divisions. Binucleate cells are found near the base, but not tip, of a fold. The fold can be considered to have a tip and a base, and cell fusions occur at the base. The folds develop in the gonad “bare zone” where sheath cells do not cover the gonad (see Fig 1A), and eversion occurs in a region with sheath cell contacts.

Several somatic cells in *C*. *elegans* are multinucleate, including some that are binucleate [4, 78]. Most of these multinucleate cells have been shown to result from cell fusions that are mediated by the fusogens AFF-1 or/and EFF-1 [79]. We found binucleate germ cells in *eff-1(ok1021)* and *aff-1(tm2214)* mutant gonads, as well as in *eff-1(ok1021) aff-1(RNAi)* and *eff-1(RNAi) aff-1(tm2214)* gonads (15–27 gonads each, Fig 11J). Thus, binucleate germ cells might result from fusions that are mediated by other, unknown fusogens. Because germ “cells” are syncytial compartments created by bending a single plasma membrane, an alternative possibility is that germ cell fusions result from simple deformations of that membrane (Fig 12A).

Although the distalmost binucleate cells in adult hermaphrodites often occur in or near the transition zone (Fig 11E), we did not find any binucleate cells in or near the transition zones in L4 hermaphrodite gonads or in adult male gonads (n = 24, 38 gonads respectively). However, we noticed that the diameter of the gonad core in an adult hermaphrodite gonad expands considerably near the region where binucleate cells first appear (Fig 11A), and that a similar expansion does not occur in L4 hermaphrodites or in adult males (S9 Fig). Moreover the amount of core expansion increases with the age of adult hermaphrodites (S9 Fig), as does the number of binucleate cells.

**Fig 13:**
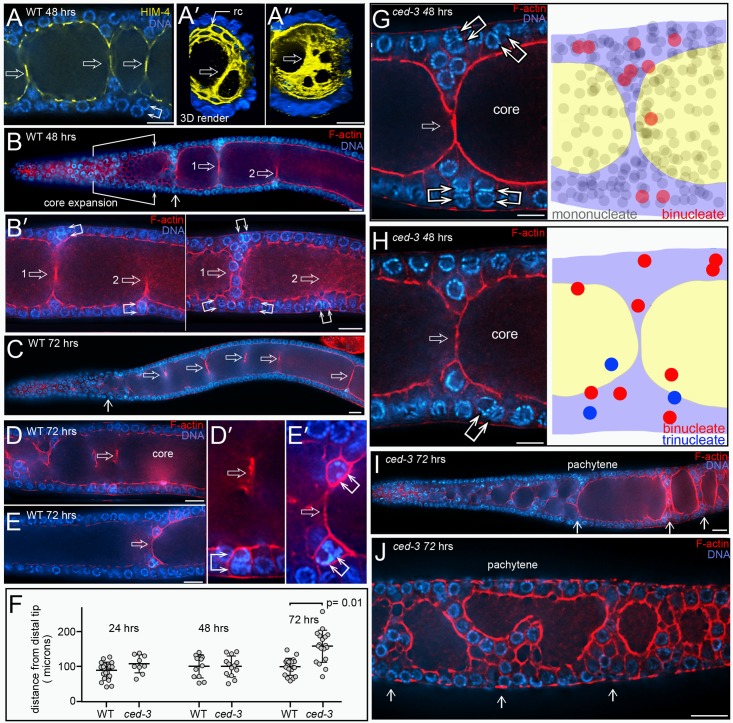
Folds/remnants and binucleate germ cells. (A) Wild-type gonad at 48 hrs. Immunostaining for HIM-4 (yellow) outlines the core, and shows membranous, anuclear material (open arrow) extending between what appear to be two sides of an incompletely resolved fold; we refer to the anuclear, membranous material as a remnant. Cells bordering the remnants often appear elongated or stretched, as shown in the panel. Panels A’ and A” show rendered views of an orthogonal projection through the gonad; panel A’ is a rope-like remnant, and panel A” is a remnant that resembles a perforated sheet. (B) Wild-type gonad showing the typical region of core expansion. A fold (vertical arrow), and two remnants (open arrows 1 and 2) persist into the early and mid-pachytene regions of the gonad, respectively. A higher magnification of the remnants is shown in panel B’ (left); note that binucleate cells appear at the bases of both remnants. The second panel in B’ (right) is a higher optical section of the same region, showing additional binucleate germ cells at the bases of the remnants. (C-E) Gonads from 72 hr wild-type adult, showing remnants (open arrows) in several regions of the gonad core. Panels D and E, and the corresponding high magnifications in panels D’ and E’, show apoptotic binucleate cells associated with the bases of the remnants. (F) Graph showing the positions of the proximal-most fold (open circles) in the indicated gonads, measured from the distal tip of the gonad; Student’s t test. For example, folds typically are cleared by 100 microns in a 72 hr wild-type adult, although remnants persist much further proximally. (G) Fold in a *ced-3(n3692)* gonad at 48 hrs. Similar to wild-type gonads, binucleate cells appear by the bases of folds. The diagram at right is a projection of every cell in this region of the gonad, overlaid on a cartoon of the middle focal plane. Note that binucleate cells appear concentrated by the fold. (H) Fold in a *ced-3(n3692)* gonad as in panel G, but containing trinucleate germ cells. (I and J) *ced-3(n3692)* gonads at 72 hrs, showing folds (vertical arrows), rather than remnants, that persist through the pachytene region.

The developmental basis for core expansion has not been described, but previous studies have noted complex arrangements of cells in this region, and described “bridges” through the core that are hypothesized to function as diffusion barriers within the gonad [80]. We examined germ cell shapes and contacts throughout the distal region of the adult gonad and in the region of core expansion, and examined the three dimensional shape of the core (S9 and S10 Figs, and S7 Video). Briefly, we found that all germ cells in this region contact the core, but the “interior” germ cells lose contact with the basal surface of the gonad (S10 Fig and legend). A simple interpretation of these results is that the germ nuclei and associated membrane domains fold and unfold like an epithelial cell layer, simultaneously deforming the gonad core. Folding/unfolding movements of epithelial cell layers are common features of animal morphogenesis, and can result from cell division, growth, or cytoskeletal changes [81]. Here, we propose that mitosis/growth within the nuclear layer of the germline syncytium causes it to invaginate or fold inward (S10 Fig and diagram in Fig 12B). Core expansion results from the unfolding or eversion of the folds, such that all germ cells establish/regain contact with sheath cells at the gonad periphery (Figs 11A and 12B, and S10 Fig). The folds in 24 hr adults are relatively small, and evert within the distal third of the gonad (S9 Fig). The folds in 48 hr adults and mated 72 hr adults are much larger and complex, and can be associated with a large-scale, helical twisting of the core (S9 and S10 Figs, and S7 Video). Some 48 hr adult gonads, and many 72 hr adult gonads, contain structures in the mid-pachytene region that appear to be incompletely resolved folds, or remnants of folds (Fig 13A–13C and S11 Fig). The remnants contain few or no nuclei, and consist of rope-like strands, or perforated sheets, of membranous material (Fig 13A). We found that binucleate cells appear to be enriched near the bases of everting folds (Fig 13B), and that apoptotic binucleate cells appear to be enriched by the remnants (Fig 13C–13E, and S12 Fig).

Binucleate cells are found at the bases of folds in *ced-3* mutant adults, similar to wild-type adults (Fig 13G and 13H, and S8 Video). *ced-3* mutant adults at 24–48 hrs appear to evert their folds as efficiently as wild-type adults, such that the mid-pachytene region contains few or no folds (Fig 13F). However, folds often persist into the mid-pachytene region of 72 hr *ced-3* adults, where most wild-type adults contain only remnants (Fig 13F, 13I and 13J, and S11 Fig). These persistent folds/remnants show a marked enrichment of binucleate cells, and include trinucleate cells that are uncommon in wild-type gonads (S12 Fig). If a trinucleate cell results from a secondary fusion of a binucleate cell, this event might be more likely when binucleate cells are not removed by apoptosis.

**Fig 14:**
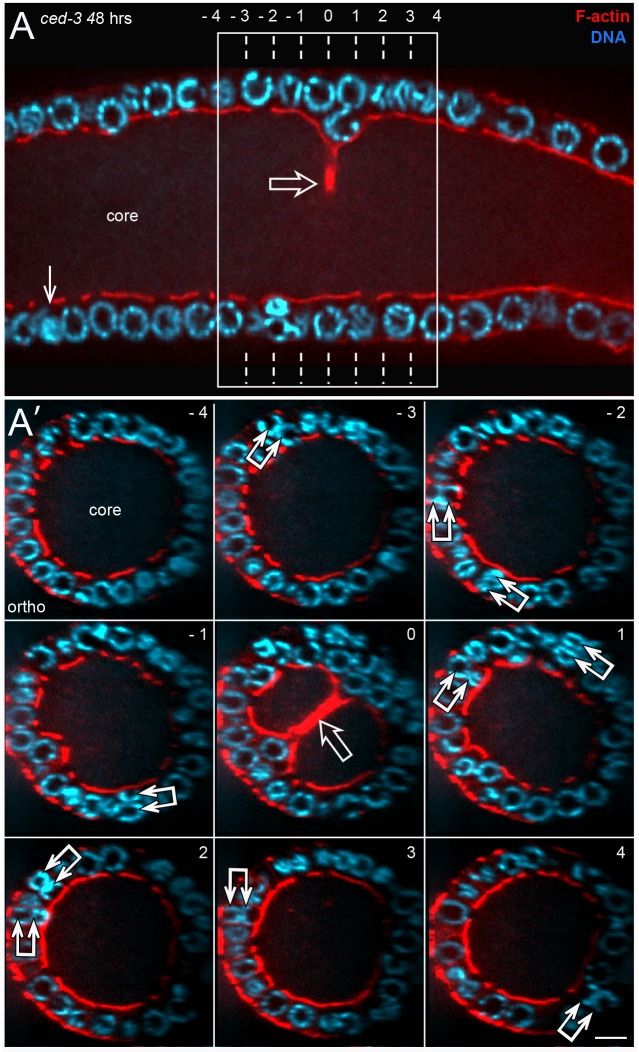
Nuclear stacking in binucleate germ cells. (A) *ced-3(n3692)* gonad at 48 hrs. The complete optical stack through the region shown contains a total of 11 binucleate cells, one of which is indicated by a vertical arrow. The remaining 10 occur with the boxed region surrounding a remnant (open arrow). (A’) Orthogonal views of numbered planes as indicated in panel A; the remnant appears in the central plane (numbered 0). Each of the 10 binucleate cells is indicated by a double arrow; note that the nuclei in each binucleate cell are stacked radially. Bar = 5 microns.

The two nuclei in a binucleate cell show a pattern of stacking, or alignment, that is correlated with the proximity of a fold or remnant. Binucleate cells near the base of a fold often contain radially stacked nuclei (Fig 13A–13D and 13G), parallel to the radial alignment of cells within a fold (diagrammed in Fig 12B). This pattern is particularly evident in orthogonal views of the gonad (Fig 14A). By contrast, binucleate cells that are not near folds or remnants typically have nuclei that align in the same plane as other germ nuclei (Fig 8D). This difference in stacking might arise if binucleate cells result from fusions between cells within a fold, and that the nuclei in a binucleate cell tend to align with other germ nuclei after the fold everts and the cells move proximally (Fig 12B).

## Discussion

Apoptosis in mammalian cells can take several hours to a day, with cell shrinkage and chromatin compaction evident at about 4 to 12 hours [82]. Apoptotic germ cells in *C*. *elegans* undergo two phases of shrinkage. The first occurs by the rapid expulsion of cytoplasm through an open ring channel, and does not involve nucleoplasmic or cytoplasmic compaction (Fig 1E). For example, we imaged PGL-1 aggregates in apoptotic germ cells that move through ring channels and into the core during cell shrinkage. F-actin levels increase at the cell periphery during the shrinkage, suggesting that actomyosin contractility drives shrinkage and the loss of cytoplasm. A second, slower phase of shrinkage begins after the ring channel closes, and is associated with both nucleoplasmic and cytoplasmic compaction. Engulfment does not appear to contribute to the first phase of shrinkage, which appears identical in both wild-type and *ced-1* mutant apoptotic cells. However, engulfment/degradation appears to accelerate the second phase of shrinkage, as compacted apoptotic cells are much more frequent in wild-type gonads than in *ced-1* gonads (23% versus 1.5%; S1 Table). Indeed, some somatic cells in *C*. *elegans* that are programmed to die occasionally survive and differentiate when engulfment is blocked [83–85].

Our TEM and live imaging studies suggest that the first phase of shrinkage expels general cytoplasmic components such as ribosomes, vesicles, and lipid droplets. By contrast, many mitochondria exit apoptotic cells before there is detectable cell shrinkage. We found that a few mitochondria exit non-apoptotic germ cells and enter the gonad core, consistent with previous studies [25]. This raises the possibility that random mitochondrial loss above a critical threshold might trigger physiological apoptosis. We consider this unlikely as a general mechanism, because we did not see appreciable variation in the amount of mitochondria in *ced-3* mutant gonads, and because apoptotic cells in *klc-1* mutants retain abundant mitochondria. Instead, we propose that apoptosis triggers mitochondrial exit.

Our results suggest that mitochondria exit apoptotic cells by specific transport along MTs. MTs in normal germ cells are oriented radially with respect to the axis of the gonad, with MT plus ends extending into the core (Fig 1D). We showed that MTs retain this radial orientation during mitochondrial exit, that exiting mitochondria are elongated parallel to the MTs, and that contacts between exiting mitochondria and MTs are visible by TEM. Finally, we showed that mitochondrial exit requires the kinesin heavy chain UNC-116/KHC, a plus-end-directed motor. Kinesin heavy chains transport diverse cargos along MTs, and some of these cargos bind directly to tetratricopeptide repeats (TPR) on kinesin light chains [86], [87], [88]. Immunolocalization studies suggest that kinesin light chains localize to mitochondria in cultured mammalian cells and in rat spermatids [89] [90], and the TPR domain of a kinesin light chain has been reported to bind a component of the mitochondrial outer membrane [91]. However, the most extensive genetic and molecular studies on mitochondrial transport have been done in neurons, where transport requires the kinesin heavy chain but not a kinesin light chain [50]. Instead, the kinesin heavy chain uses an adapter (TRAK/Milton) to bind MIRO on the mitochondrial outer membrane. We showed that mitochondrial exit from apoptotic cells requires the kinesin light chain KLC-1, but does not require MIRO-1/MIRO. Thus, *C*. *elegans* germ cells might use a novel mechanism to transport mitochondria during apoptosis, or the role of kinesin could be indirect. For example, studies in both *C*. *elegans* and *Drosophila* suggest that kinesin heavy chain can have roles in patterning and sliding MTs [92, 93].

Why do apoptotic cells use a special mechanism to shed mitochondria, rather than utilize the bulk flow associated with cell shrinkage? Mitochondria can be long relative to germ cell diameters, and often coil within the cell. If bulk flow is driven by constriction of the cell periphery, as seems likely, coiled mitochondria might become entangled during exit and difficult to remove. For example, apoptotic cells in *klc-1* mutants shrink, but lose only a small and variable fraction of their mitochondria. An additional, more interesting possibility is that selective transport during apoptosis allows mitochondrial quality to be assessed, such that defective mitochondria are detained and eliminated with the apoptotic cell. Once a mitochondrion exits a germ cell, it enters the gonad core where non-selective flow transports materials directly into oocytes (Fig 1A); for example, flow in the gonad core can transport inert materials such as latex beads or oil droplets at the same rate as cytoplasmic organelles [25]. Thus, any defective mitochondria that entered the core would likely be incorporated into oocytes. Apoptosis is a common fate for germ cells in animals, including humans, and one proposed role for germ cell apoptosis is the removal of gametes with defective mitochondria [94]. Although most apoptotic germ cells in *C*. *elegans* expel all of their mitochondria into the core, apoptotic cells often retain one or a couple of mitochondria, and some of these mitochondria have abnormal shapes. Thus, it will be interesting in future studies to determine whether the residual mitochondria in apoptotic cells are defective, and whether mitochondria accumulate mutations in apoptosis-defective strains.

### Maintaining the integrity of apoptotic germ cells

Although apoptotic cells in wild-type gonads are rapidly engulfed, degraded and cleared, apoptotic cells in engulfment-defective mutants appear to persist for long periods of time. We found that the execution phase of germ cell apoptosis initiates major changes in the cytoskeleton (Fig 1E), but that these changes are interrupted at various stages by engulfment and degradation. We propose that these changes contribute to the persistence of apoptotic cells in gonads where engulfment is blocked or delayed. Although these cytoskeletal changes might not be essential for young animals grown in the laboratory with abundant food, where engulfment is immediate, it is possible that stress or environmental factors can impact the rate of engulfment and degradation.

Many animal cells depolymerize their MTs early in the execution stage of apoptosis, but some types of apoptotic cells reform a novel, cocoon-like array of cortical MTs [95]. Among other functions, this array may maintain cell morphology and the integrity of the plasma membrane. We showed that *C*. *elegans* apoptotic germ cells have radially-aligned MTs during mitochondrial exit and cell shrinkage, similar to the MT array in non-apoptotic cells. The MT pattern changes markedly after ring channel closure, and resembles the cocoon or cortical arrays described in other types of apoptotic cells. MTs are intrinsically dynamic polymers, but can be stabilized by specific tubulin modifications such as acetylation or polyglutamylation, or by MT-binding proteins such as Tau [96, 97]. We found that most or all of the MTs in the cocoon-like array appear to be stabilized: They do not show dynamic EB1::GFP comets (Fig 4D), and they are resistant to the depolymerizing agent nocodazole (Fig 4F). In future studies, it should be possible to identify and disrupt MT-stabilizing modifications or accessory proteins, and thus test the function of the cocoon array. Animal cells nucleate MTs from centrosomes (centriole pair plus pericentriolar material), or from non-centrosomal sites such as the plasma membrane. MTs nucleated by centrosomes can form a radial array around the centrosome, or the MTs can detach from the centrosome and move elsewhere in the cell [98]. Centrosomes in the *C*. *elegans* gonad are thought to become inactive as MT-nucleating centers after germ cells exit mitosis [57, 58], although centrioles persist through the pachytene stage [58]. We did not observe MTs associated with centrioles in non-apoptotic germ cells, as expected, but found multiple apoptotic cells with MTs in close proximity to centrioles. Studies on somatic cells in *C*. *elegans* suggest that the MT-nucleating function of centrosomes can switch reversibly between “off” and “on” states [99], so it will be interesting to determine whether a similar switch occurs during germ cell apoptosis.

The actin cytoskeleton also changes markedly after apoptotic cells shrink and close. Actin becomes concentrated in giant microfilament bundles that resemble cofilin-actin rods in other systems. Cofilin-actin rods have not been reported previously in normal *C*. *elegans*, but rods have been induced experimentally in *C*. *elegans* muscle cells by transgenic over-expression of a muscle-specific UNC-60/cofilin isoform [100]. Cofilin-actin rods consist of cofilin-saturated actin microfilaments in a 1:1 ratio of cofilin to actin [59]. Despite containing large numbers of actin microfilaments, cofilin-actin rods show little or no staining with phalloidin because cofilin alters the phalloidin binding site between actin monomers [101] [102]. Consistent with those characteristics, the rods in the *C*. *elegans* apoptotic germ cells stain reproducibly with antibodies against UNC-60/cofilin or actin, but show highly variable or no staining with phalloidin. Because the cofilin-actin rods identify apoptotic cells independent of engulfment, they should be a useful marker for future studies on germ cell apoptosis: Most of the reporters currently used to study germ cell apoptosis in *C*. *elegans*, such as acridine orange, SYTO-12, and sheath-specific GFP expression, do not identify apoptotic cells in engulfment-defective mutants [103].

Cofilin-actin rods are of clinical interest as they form inappropriately in several myopathies and neurodegenerative diseases such as Alzheimer’s and Huntington’s disease [60] [62] [104] [105]. The rods can be induced in some types of cells by a variety of stresses that include ATP depletion. Low ATP levels appear to trigger cofilin dephosphorylation at a conserved Ser-3 residue, thereby converting cofilin from an actin-depolymerizing protein into an actin-stabilizing protein. Because cofilin-actin rods lock actin into a stable polymer, rod formation has been proposed as a mechanism to prevent actin dynamics from depleting ATP in stressed cells. Mitochondria are the principle source of ATP, and we showed that mitochondria exit apoptotic cells before the cofilin-actin rods form. We expect mitochondrial loss to deplete ATP, but we do not know whether additional ATP is generated in the apoptotic cells by aerobic glycolysis. Indeed, we consider it an interesting and unresolved question when non-engulfed, apoptotic cells should be considered as “dead”. We found that azide treatment was sufficient to induce cofilin-actin rods in the gonad core, particularly in the mitotic region, but did not induce cofilin-actin rods within the germ cells. These results suggest that rod formation in apoptotic cells does not result solely from the loss of mitochondria. Interestingly, we found that the level of cytoplasmic UNC-60/cofilin appears to increase in apoptotic cells prior to rod formation; some mRNAs are known to be translated in apoptotic cells in other systems, despite an otherwise general repression of protein synthesis [106].

As apoptotic germ cells undergo the second, slow phase of shrinkage, the cytoplasm appears to compact, and bumps or ridges appear at the cell surface above the coflin-actin rods (Fig 1E). Apoptotic cells in other systems commonly form actomyosin-dependent surface blebs, and the blebs can be precursors of apoptotic bodies during cell fragmentation. Recent studies have shown that blebs may also contribute to the recognition and clearance of apoptotic cells; “eat me” signals can be concentrated in the blebs, and inhibiting bleb formation inhibits corpse removal [107]. It remains to be determined whether the cofilin-actin rods in *C*. *elegans* contribute to interactions between apoptotic cells and the phagocytic sheath cells, but most wild-type apoptotic cells appear to have only rudimentary, or no, rods when they are engulfed.

### Physiological apoptosis includes the removal of binucleate germ cells

We found that gonads contain binucleate germ cells at all stages of adult development, and propose that these cells are removed as part of the process called physiological apoptosis. For example, the binucleate cells form under standard culture conditions, and form independent of genes that are essential for stress-induced apoptosis. Computational modeling has suggested that germ cells could be selected for apoptosis randomly, or on the basis of size [30]. The selection process cannot be entirely random, as 100% of the binucleate cells are selected for removal before they develop into oocytes. Although mononucleate apoptotic cells appear to be the same size as neighboring cells, binucleate apoptotic cells are, indeed, slightly larger than their neighbors. In future studies, it should be possible to discover how binucleate cells are selected for apoptosis by screening for mutants with binucleate oocytes.

Physiological apoptosis is usually described as occurring only in late pachytene-stage germ cells, near the gonad loop. The majority of apoptotic cells are found near the loop, but binucleate apoptotic cells occur in the mid-pachytene region, and occasionally in the early-pachytene region. Most binucleate cells appear to form near the transition zone, but we do not know when they first become committed to apoptosis. We found that about 20–30% of all apoptotic cells in engulfment-defective adults are binucleate, and that the total number of binucleate cells increases markedly with adult age. Binucleate apoptotic cells are visible in wild-type gonads with engulfment-dependent reagents that are commonly used in *C*. *elegans* studies. However, binucleate apoptotic cells might have escaped previous scrutiny for two reasons. First, a single, engulfed binucleate cell might be interpreted as two engulfed cells, because sheath cells often contain two or more apoptotic cells. Second, the binucleate cells originate distally, and then are removed sequentially by apoptosis as they move proximally. This means that wild-type gonads rarely contain more than one engulfed binucleate cell. By contrast, multiple cells near the loop often undergo apoptosis simultaneously (S3 Video).

The existence of different types of apoptotic cells, binucleate and mononucleate, might contribute to some of the observed genetic complexity of physiological apoptosis. For example, VAB-1/EphR signaling has been shown to promote apoptosis, but apoptosis is reduced by only 50% in *vab-1* mutants [108]. Thus, it might be informative to examine binucleate apoptotic cells in some of these mutants. Several lines of evidence suggest that MAPK signaling has a role in physiological apoptosis, but it is not clear whether that role is direct or indirect; for example, germ cells require MAPK signaling to exit the pachytene stage of meiosis [5, 109, 110]. Most apoptotic cells occur after the peak of activated MAPK, as inferred by immunostaining for dpMPK-1, while most apoptotic binucleate cells occur before the peak. Thus, a simple possibility is that apoptosis of binucleate cells is independent of MAPK, while MAPK functions in the apoptosis of germ cells near the gonad loop. However, we found that most apoptotic germ cells in *nos-3;mpk-1(null)* double mutants contain a single nucleus. The observation that apoptotic cells also are present in *cep-1(RNAi); nos-3; mpk-1(null)* gonads suggests that they do not result from the CEP-1-dependent, DNA-damage pathway. However, we cannot exclude the possibility that the deaths result from some other MAPK-independent stress that is not indicative of physiological apoptosis.

We showed that *ced-3* gonads contain many binucleate, and some trinucleate, germ cells, yet few of these ever reach the proximal half of the gonad to differentiate as oocytes. Each gonad arm contains about 1000 germ nuclei, but hermaphrodites only produce about 150 sperm. Because binucleate cells originate toward the distal end of the gonad in young adults, they trail a large queue of mononucleate oogonia whose fertilization depletes the store of self-sperm. Thus, ovulation normally ceases in *ced-3* mutants before most binucleate germ cells reach the proximal half of the gonad. Ovulation can resume after *ced-3* adults are mated, and a few binucleate germ cells enter the proximal gonad where they become enlarged binucleate oogonia, and occasionally binucleate oocytes. However, mating allows only a limited extension of *ced-3* oogenesis: Without apoptosis, the excess cells in the gonad loop fail to intercalate into a single line. As these extra oogonia take up core cytoplasm simultaneously, the core narrows and then collapses. Our results suggest that the rare binucleate germ cells that reach the proximal half of the gonad and become oocytes have the potential to develop into viable, triploid embryos. Although apoptosis prevents any binucleate germ cells from becoming oocytes in wild-type animals cultured under optimal laboratory conditions, we speculate that *C*. *elegans* might exploit the potential for making polyploid progeny in certain suboptimal environments [111].

### Binucleate germ cells and growth of the germline syncytium

Our results suggest that binucleate cells form in conjunction with folds that develop in the gonad syncytium. Epithelial folding is a common event in animal morphogenesis; for example, the *Drosophila* leg imaginal disc is an epithelial sac that develops interior folds as cells proliferate and grow, and then everts during morphogenesis [112]. Although the *C*. *elegans* gonad is a syncytium, most germ nuclei have a layer-like organization: They share a common polarity, they show a smooth, layer-like deformation as they pass beneath sheath cell bodies, and they bend like a layer as they move around the gonad loop. As proliferation/growth increases cell density in the distal gonad, the nuclear layer appears to fold inward, often creating a helical twist that can be seen by the complementary, 3D shape of the core (S7 Video). Germ cell division axes are not, in general, aligned along the radial axis of the gonad, so we imagine that folding results from indirect, steric forces associated with division and growth. Folding occurs in a “bare” region of the gonad that is not covered by sheath cells, and eversion begins in a region lined with sheath cells (Fig 1A). Thus, it is possible that the absence of germ cell-sheath cell adhesion facilitates folding, and that the resumption of sheath adhesion promotes eversion.

Binucleate germ cells appear to form by the fusion of two postmitotic germ cells, rather than by incomplete cytokinesis: Binucleate cells are not found in the mitotic regions of hermaphrodite gonads, and the pattern of EdU labeling in binucleate cells is not indicative of sister nuclei. Moreover, if a binucleate cell divided it would be expected to form a tetrapolar spindle, resulting in aneuploid daughter cells. We propose that binucleate cells instead arise during the eversion of folded regions of the germline syncytium, and are subsequently removed by apoptosis. This proposal is based on several correlations: First, the number of binucleate cells increases with adult age, as does the size and complexity of the folds. Conversely, binucleate cells are not found in males or L4 hermaphrodites, which have only small folds in the germline syncytium. Second, binucleate cells are first detected in the same region that the folds begin to evert, and binucleate cells appear clustered around these folds. Binucleate cells similarly cluster around folds that persist into the mid-pachytene region. Third, binucleate cells typically have radially stacked nuclei, which might result from the fusion of radially stacked cells within a fold (Fig 12B). Finally, trinucleate germ cells are rare in wild-type gonads, but occur frequently in *ced-3* gonads; most of the trinucleate germ cells occur near folds that persist into the mid-pachytene zone. The latter result suggests that fusion events can occur outside of the transition zone, and thus are not dependent on cytoskeletal changes associated with meiotic chromosome pairing and synapsis [113].

In general, the fusion of two separate cells requires a change in membrane topology, and is mediated by special fusogens such as EFF-1 and AFF-1 in *C*. *elegans*. However, neither *eff-1* nor *aff-1* appear necessary for the formation of binucleate germ cells. Because germ “cells” are compartments created by bends in a single plasma membrane, fusions between adjacent compartments might result from small changes in membrane bending, without altering topology (Fig 12A). The plasma membrane deformations that define the compartments are maintained normally by compartment-compartment adhesion, and by adhesion associated with the ring channel. For example, several actin-binding proteins such as ANI-2/Anillin are enriched by the ring channels, and depletion of ANI-2 creates multinucleate germ cells [114]. Thus, germ cell fusion could involve regulatory pathways that specifically downregulate or modify adhesive proteins, or fusion might result from non-specific stretching forces during fold eversion.

Finally, we suggest that the formation of binucleate cells might contribute to gonad homeostasis, by providing a measure of whether the number of distal germ cells is “appropriate” for the size and state of the gonad. The gonad is like a conveyor belt, where the addition of cells at the distal end (by mitosis) is likely coordinated with the removal of cells at the proximal end (by ovulation). We propose that large folds form when the rate of addition is faster than cells move away from the distal end. In addition, conditions that decrease the diameter of the core, such as starvation, increase the relative size of existing folds. For a given core diameter, we imagine that large folds will be more difficult to evert than small folds, and will generate correspondingly more binucleate cells. Because these binucleate cells will be removed by apoptosis, they provide a system to reduce excessive numbers of germ cells before they reach the loop region, where they might interfere with intercalation and oogonial expansion.

## Methods

### Worm stains and culture conditions

General nematode culture was as described [116]; all strains were derived from the wild-type Bristol strain N2, and all animals were grown at 20°C. The mutant alleles and fluorescent reporters used in this study were as follows and obtained from the CGC unless noted otherwise:

**LG I:** *fog-1(q253ts)*, *cep-1(gk138)*, *ced-1(e1735)*, **LG II:** *fzo-1(tm1133)*, *pch-2(tm1458)*, *nos-3(oz231)*. **LG III**: *mpk-1(ga117)/qC1*::*GFP*, *nos-3(oz231)*, *ced-4(n1162)*, *ced-7(n1892)*. **LG IV:** *ced-2(1752)*, *miro-1(tm1966)* (a gift from Takao Inoue*)*, *ced-5(n1812)*,*ced-3(n717)*, *ced-3(n3692)*. **LG V:** *egl-1(n1084n3082)*. The strain *nos-3(oz231)II; mpk-1(ga117)/qC1*::*GFP III* was a gift from Tim Schedl [74].

**BT24**: *rhIs23*; [*GFP*::*HIM-4*], **JJ2101**: *zuIs242;* [*nmy-2p*::*PGL-1*::*GFP + unc-119(+)*], **JJ1477**: [*nmy-2p*::*ACT-1*::*GFP + unc-119(+)*] gift from Ed Munro, **MD701**: *bcIs39*; [*lim-7p*::*CED-1*::*GFP + lin-15(+)*] gift from Bob Horvitz, **JJ2208** (*nmy-2p*::*PGL-1*::*GFP*) [25], **JJ2212**: [*nmy-2p*::*PGL-1*::*mRFP*] [25], **JJ2586** (COX-4::GFP) this study, **TH61**: *ddIs36*; [*pie-1p*::*GFP*::*SAS-5 + unc-119(+)*], **OC95**: *bsIs2*; [*pie-1*::*GFP*::*SPD-2 + unc-110(+)*], **OD70**: ltIs44; [*pie-1p*::*mCherry*::*PH(PLC1d1)*], **OD1359**: *itSi716*; [*pmex-5*::*EBP-2*::*GFP + unc-119(+)*] gift from Karen Oegema [56], **WH223**: *ojls*9; [*ZYG-12*::*GFP + unc-119(+)*]

### Antibodies

Antibodies against the following proteins were used for this study:

Actin (C4, kMillipore), ATP synthase beta (ab14730 Abcam), Cytochrome c oxidase subunit 1 (ab14705 Abcam), dpMPK (M8159, Sigma), GFP (Wako), HIM-4 (gift from Bruce Vogel) [117], NPP-9 [36], PGL-1 (gift from Susan Strome) [37], alpha-Tubulin (YOL3/4 Abcam), UNC-60/cofilin (gift from Shoichiro Ono) [61].

### Immunostaining

All dissections were done on taped glass microscope slides prepared by sticking invisible tape (single layer, matte finish) to a glass microscope slide, then heating the slide to 300°C for about 10 mins; the edges of the tape should appear slightly melted. The slides were wiped briefly with 95% EtOH, then dH20, before use. A drop of 30–50 ul of M9 buffer was placed on the taped slide, and 20–30 worms were picked from a culture plate directly into the drop. The worms were agitated briefly to remove any residual bacteria, and washed in two changes of M9 buffer. The M9 buffer was removed and replaced with either 30 ul of gonad buffer 1: [83 mM Hepes, pH 6.9, 90 mM NaCl, 5.7 mM KCL, 2 mM MgCl_2_] or 30 ul of gonad buffer 2: [48% Leibovitz L-15 (GIBCO), 9.7% Fetal Calf Serum (GIBCO), 1% sucrose, 2 mM MgCl_2_]. Gonads were released by cutting the worms with a scalpel blade just behind the pharynx, and the total dissection time for a group of animals was 2 mins or less.

To stain UNC-60/cofilin plus actin, gonads were dissected in 30 ul of gonad buffer 1. An additional 30 ul of 2X FIX: [5% formaldehyde (SIGMA) + 25 mM HEPES (7.4), 40 mM NaCl, 5 mM KCL, 2 mM MgCl2] was added, and the gonads were fixed for 45 secs. During the fixation, excess fixative was removed and at 45 secs the slide was inverted and dipped into a glass dish containing MeOH at room temperature. The gonads were allowed to fix in MeOH for 10 mins. The excess MeOH was removed and replaced with two changes of 2% Tween-20 in PBS (pH 7.2) for 5 mins each, then PBS for 5 mins. The gonads were transferred in PBS to teflon-coated, multi-well test slides (Tekdon) for immunostaining. Staining in primary antibody was overnight at 4°C, staining in secondary was for 1 hr at 37°C. For mounting, the coverslips were supported with 22 micron glass beads (Whitehouse Scientific) for 24 hr adults, or 28 micron glass beads for 48 and 72 hr adults.

For MT plus mitochondrial staining, gonads were dissected in 30 ul of gonad buffer 2. An additional 30 ul of 2X fixative was added and the gonads were fixed for 30 mins at room temperature. The fixative was removed and the gonads incubated with 0.3% Triton X100 (Sigma) in PBS (pH7.2) for 15 mins at room temperature. The gonads were washed with PBS several times before immunostaining. Nocodazole treatment and immunostaining for stable MTs was as described [29]. For other immunostaining experiments, gonads were dissected in gonad buffer 1 and fixed for 15 mins.

### EdU labeling of replicating DNA

Labeling of DNA with EdU was essentially as reported [118] and modified by T. Schedl http://genetics.wustl.edu/tslab/protocols/edu-labeling-and-staining/, except worms were incubated by soaking in the EdU solution for 45–60 mins. Worms were processed immediately after soaking (Fig 11F), or were removed and allowed to develop on culture plates without EdU for 17–30 hours (Fig 11G–11I). Labeled worms were fixed as for UNC-60/cofilin. Only 1–3 of the most distal apoptotic cells with cofilin-actin rods were scored in each gonad; this method of scoring should enrich for germ cells that were in the mitotic zone, which is the most distal region of the gonad, at the time of labeling.

### Chromosome/nuclear counts in oocytes

Nuclear counts in oocytes were scored in intact animals, rather than in dissected gonads, because of concern that dissection might create a low, artificial background of fused oocytes. Despite this concern, we did not observe binucleate oocytes in any dissected, wild-type gonads prepared for immunostaining in other experiments. For staining intact adults, worms were rinsed from culture plates with M9, then concentrated by settling for 10 mins. After removing the excess liquid, Carnoy’s fixative (60% EtOH, 30% CHCl_3,_ and 10% glacial acetic acid) was added to the worms. The worms were allowed to settle briefly, and the excess fixative was removed and replaced by fresh fixative. After fixation overnight at 20°C, the fixative was removed by several washes with 2% Tween-20 in PBS (pH 7.2) and then with PBS alone. The fixed worms were soaked in PBS for two hours for rehydration, then stained with DAPI (4’,6’-Diamindino-2-phenylindole, Sigma) for 2 hours before mounting.

### Live imaging

Delta T dishes (Bioptechs) were used for live imaging experiments. 20 ul of M9 was pipetted into a dish, and about five worms were added to the drop. The excess M9 was removed and replaced with 20 ul of 2mM tetramisole hydrochloride (L9756, Sigma). After 15–20 mins, the excess liquid was removed and a 1cm X 1cm slab of 2% agarose (15510027, Invitrogen) containing 20 mM serotonin (H7752, Sigma) and 2 mM tetramisole was gently lowered on the worms. A small O-shaped piece of filter paper was placed on the lid, and wetted with a few drops of M9. The lid was inverted onto the dish after shaving off the lid spacers to create a sealed chamber.

Time-lapse movies were acquired with a Hamamatsu C9100 camera on a Nikon TE-2000 inverted microscope equipped with a Yokogawa CSU-10 spinning disk; image acquisition was with Volocity 5.3.3 (Improvision). Orthogonal projections of optical Z-stacks were generated and analyzed either with Volocity software or with ImageJ [119, 120].

Widefield images were acquired with a Nikon Eclipse 90i upright microscope using 40X (Plan Fluor, 1.4 NA) and 60X (Plan Apo VC, 1.4 NA) objectives and a Retiga-4000DC camera (QImaging). Other images were acquired with a DeltaVision microscope and processed using deconvolution software (Applied Precision). Images were exported to Adobe Photoshop for contrast/brightness adjustments, reorientation and cropping.

### Electron microscopy

Gonads were processed for electron microscopy as described [29]. Grids were viewed with a JEOL JEM-1230 transmission electron microscope and photographed with a Gatan UltraScan 1000 CCD camera. For the data in S1 Table, the sections chosen for analysis were separated by at least 4 microns to ensure apoptotic cells were only scored once. Sections were screened at low magnification for apoptoic cells that appeared to be cut through the center of the nucleus; these cells were then scored at high magnification.

### Transgene construction

COX-4 (W09C5.8) was tagged at the C-terminus with EGFP using genome editing methods described in [121]. The guide sequence CGGTGCACCGACACACTACG was inserted into pDD162 using the NEB Q5 Site-Directed Mutagenesis Kit (E0554S) and the primers CGTAGTGTGTCGGTGCACCGGTTTTAGAGCTAGAAATAGCAAGT and CAAGACATCTCGCAATAGG. EGFP template plasmid was generated by digesting pJJR82 with AvrII/ClaI and using the NEBuilder HiFi DNA Assembly Master Mix (E2621S) to insert a 5′ homology arm amplified from *C*. *elegans* genomic DNA with ACGTTGTAAAACGACGGCCAGTCGCCGGCATTGGCTCGTCAAATGATGCT and CATCGATGCTCCTGAGGCTCCCGATGCTCCCTTCCACTTCTTGTTCTCGTAAT and a 3′ homology arm amplified with CGTGATTACAAGGATGACGATGACAAGAGATAAAATATAGAGATTCAGCAGAAA and GGAAACAGCTATGACCATGTTATCGATTTCTATCGAAAAGTGCTTCCACA. pJJR82 was a gift from Mike Boxem (Addgene plasmid # 75027), pDD162 (Peft-3::Cas9 + Empty sgRNA) was a gift from Bob Goldstein (Addgene plasmid #47549) [122], pGH8 –pRAB-3::mCherry::*unc-54*utr (Addgene plasmid #19359), pCFJ90 –P*myo-2*::mCherry::*unc-54*utr (Addgene plasmid#19327) and pCFJ104 –P*myo-2*::mCherry::*unc-54*utr (Addgene plasmid #19328) were gifts from Erik Jorgensen [123].

## Supporting information

S1 Table TEM analysis of 48 hr wild-type gonads and *ced-1(e1735)* gonads. Germ cells were scored in the late-pachytene and loop regions of the gonad. A sheath vesicle enclosing an apoptotic cell was scored as a phagosome if the separation between the vesicle membrane and the plasma membrane of the apoptotic cell was 0.2 microns or less, and scored as a phagolysosome if the separation was greater than 0.2 microns (see for examples S2 Fig). The percentage of cells contained in a phagolysosome was scored relative to total germ cells, and for total engulfed germ cells (parentheses). Irregularly-shaped nuclei with deep depressions were scored as lobed; see for example S2 Fig. Cells with perforated nuclear envelopes usually appeared to be necrotic; they were typically larger than cells with dark, compacted cytoplasm, and had far less ribosomal density.
(DOCX)

S1 Fig Live imaging of apoptosis. (A) Video sequence showing PGL-1 loss, cell shrinkage, engulfment, and the development of DIC refractility in a wild-type apoptotic cell (X). The gonad expresses reporters for PGL-1 (red, PGL-1::RFP) and for the nuclear envelope (green, ZYG-12::GFP). PGL-1 is expressed only in germ cells, but the PGL-1::RFP transgene used here has a heterologous promoter that drives additional, ectopic expression in the sheath cell cytoplasm. This dual expression allows the loss of PGL-1 from P granules in an apoptotic cell to be tracked simultaneously, and in the same channel, with the engulfment of that same cell by the sheath. At t = 0 mins the apoptotic cell (X, dashed outline) appears similar in size to adjacent, non-apoptotic germ cells, and has a similar level of PGL-1 on P granules (double arrow). By 15 mins, most of the PGL-1 has disappeared from the apoptotic cell, and sheath cell protrusions (arrowheads) have nearly engulfed the apoptotic cell body. The sheath protrusions outline the apoptotic cell and reveal the amount of shrinkage. The general DIC appearance of the apoptotic cell resembles that of non-apoptotic cells until about 79 mins, but the apoptotic cell becomes refractile by 96 mins as it is degraded within the sheath. (B) Video sequence of PGL-1 loss and cell shrinkage of a *ced-1(e1735)* apoptotic cell (X). The gonad expresses a reporter for PGL-1 (red) as above. However, because sheath cells will not engulf apoptotic cells in this mutant, a germ cell specific membrane reporter (also red) is included to track cell outlines. PGL-1 begins to diminish at about 12 mins, coincident with the start of cell shrinkage. (C-I) These recordings address whether cells destined for apoptosis differ in size from non-apoptotic cells. The sequence of panels (C-I) is arranged in a distal to proximal order through the gonad, and shows the gradual and uniform increase in cell sizes as germ cells approach the gonad loop. Each panel shows two timepoints taken from live recordings of *ced-1(e1735)* gonads expressing the reporters as listed. The lower frame identifies cells that underwent apoptosis during the recording (X), and adjacent, non-apoptotic cells (numbered). The upper frame shows the same cells at an earlier timepoint, and indicates the elapsed time between the start of the recording (t = 0 mins) and the first timepoint that loss of PGL-1 and/or cell shrinkage was observed in the apoptotic cell. For example, none of the germ cells shown in panel C decreased in size from t = 0 mins until t = 30 mins (shown), but the apoptotic cell began to shrink at the following timepoint. Because the initiating step of apoptosis has not been defined, we do not know whether the cells labeled X in the first timepoints are already committed to apoptosis, or are instead pre-apoptotic cells. Thus, these experiments show directly that the sizes of apoptotic cells do not differ from neighboring cells for at least 38 minutes prior to shrinkage. Moreover, we did not observe a subpopulation of atypically large cells in any of our recordings of germ cells near the loop of the gonads (compare cells within the individual fields shown at the t = 0 mins timepoints). (J) Shrinkage of an apoptotic cell (X) without apparent nuclear shrinkage. Video sequence of a wild-type gonad labeled as in panel A, but using a different reporter for the nuclear envelope (green, NPP-9::GFP). The perimeter of the apoptotic cell (dashed line) at t = 0 mins was inferred by overexposing the red channel to show cytoplasmic PGL-1; the perimeter at 14 mins is indicated by the enveloping sheath cell (arrowheads). Bars = 5 microns. Fluorescent strains: (A) JJ2212 + WH223, (B) JJ2212 + OD70 + WH223, (C,E) OD70, (D, G, I) OD70 + JJ2208, (F, H) OD70 + JJ2212, (J) JJ2212 + JH2184.
(TIF)

S2 Fig TEM of apoptotic germ cells. (A-A’) TEM micrograph of a wild-type gonad, comparing a normal germ cell (left) with an apoptotic cell (X). Arrowheads in panel A’ indicate the nuclear envelope (white) and plasma membrane (yellow) of the apoptotic cell, and the sheath cell process (green). The apoptotic cell has a smooth, round nucleus and has a ribosome density comparable to the non-apoptotic cell and to the core. The sheath cell has surrounded the apoptotic cell, but the sheath cavity/phagosome (purple) has not expanded into a phagolysosome. This combination of features suggests that the apoptotic cell is at a relatively early stage after shrinkage. (B-B’) The image shows three apoptotic cells (X1, X2, and X3) in a wild-type gonad; parts of two non-apoptotic cells are visible to the left of the panel. All three apoptotic cells are in a large phagolysosome (purple) within a single sheath cell. Panel B’ shows the apoptotic cells at higher magnification. Note that the densities of the cytoplasm and nucleoplasm in X2 appear greater than in the non-apoptotic cell to the left, and that the densities in X1 appear greater than in X2. Examination of semi-serial sections through this gonad showed that the X1 cell is smaller in size than the other two apoptotic cells. Bars = 1 micron.
(TIF)

S3 Fig Mitochondrial exit from apoptotic cells. (A-D) TEM micrographs of wild-type, presumptive apoptotic, germ cells that have open ring channels but that appear to be shrinking; the cells are shown in the order of decreasing cytoplasmic volume. Arrowheads indicate the nuclear envelope (white) and the plasma membrane (yellow) of the apoptotic cell, and sheath cell processes (green) are indicated where present. Note that the cells have lost all or most of their mitochondria (cartoon at bottom, labeled as for S2 Fig). Note also that the nuclei are close to the basal pole of the cells; live imaging showed that apoptotic nuclei shift basally as they lose mitochondria. (E) Example of a closed and engulfed, wild-type apoptotic cell that retained a mitochondrion (white arrow). Bars = 1 micron (A-E).
(TIF)

S4 Fig Centrioles in apoptotic and non-apoptotic germ cells. (A-D) TEM micrographs of centrioles in wild-type germ cells; panels A and B show non-apoptotic cells, and panels C and D show apoptotic cells. A centrosome consists of the paired centrioles (black arrows) and the associated pericentriolar material (PCM); the PCM typically appears in TEM micrographs as a zone of ribosome exclusion surrounding the centrioles. The paired centrioles can be aligned parallel (panel B), or orthogonally (panel A), and can have variable spacing. No MTs are visible near the centrioles in the non-apoptotic cells shown in panels A and B, but MTs (white arrows) are near the centrioles in both apoptotic cells (panels C and D); panel C shows a cross-sectional profile of an MT, and panel D shows a longitudinal profile of an MT. Note also that panel D is an example of a binucleate apoptotic cell. (E, F) Centrioles persist in apoptotic germ cells; panel E is a *ced-1(e1735)* gonad and panel F is a wild-type gonad. The gonads express transgenic reporters for centrioles (SPD-2::GFP in panel E and SAS-5::GFP in panels F, G) and the centrioles are visualized here by immunostaining for GFP (green). Apoptotic cells are indicated by X, and examples of non-apoptotic cells are numbered. Centrioles were observed in 19/19 apoptotic cells stained for SPD-2::GFP, and in 5/6 apoptotic cells stained for SAS-5::GFP. Panel G shows a field of non-apoptotic cells, including a non-apoptotic binucleate germ cell (double arrow). Note that the binucleate germ cell contains two pairs of centrioles (shown at higher magnification in panel G”). Panel G’ is a projection through the optical stack of field, and shows that all other germ cells contain a single pair of centrioles. Bars = 50 nm (A-D), 2.5 microns E-G. Reporters: (E and G) OC95, (F) TH61.
(TIF)

S5 Fig Microfilament bundles/ actin rods in apoptotic cells. TEM micrographs showing microfilament bundles (green arrows) in *ced-1(e1735)* apoptotic germ cells (A-C) and in wild-type apoptotic cells (D-F). Arrowheads indicate the nuclear envelopes (white) and the plasma membranes (yellow) in the apoptotic cells. The microfilament bundles often appear to bridge the nuclear envelope and the plasma membrane, as shown in panel B. Microfilament bundles in wild-type apoptotic cells (panels D-F) are comparatively small, and appear only in cells that appear to be at advanced stages of apoptosis. Panel F shows an example of a small microfilament bundle associated with a small bump or ridge in the surface of a wild-type apoptotic cell. Bars = 1 micron (A, D), 200 nm (B, D’, E, F).
(TIF)

S6 Fig TEM micrographs of binucleate germ cells. (A-D) TEM micrographs of binucleate germ cells, ordered in a developmental sequence. Arrowheads indicate the two nuclear envelopes (white) and the plasma membrane (yellow) of the binucleate cell. The binucleate cell in panel A is not engulfed and has an appreciable volume of cytoplasm, but appears to be at an early stage of apoptosis because the nuclei are shifted basally (down) and the cytoplasm appears to lack mitochondria (see text). The binucleate cell in panel B has little cytoplasm and is nearly engulfed, but retains an open ring channel (rc). The binucleate cell in panel C is fully engulfed. Panel D shows a binucleate apoptotic cell in a *ced-1(e1735)* gonad with several microfilament bundles, as shown at higher magnification in the inset. Bars = 1 micron A-D, 200 nm (D’).
(TIF)

S7 Fig Binucleate germ cells undergo apoptosis before the peak activation of MAP kinase. (A-F) The panels show several examples of *ced-1(e1735)* gonads at 48 or 72 hrs, as indicated, that were immunostained for activated MAPK (red, anti-dpMPK-1). The majority of apoptotic cells (brackets) occur after (on the proximal side of) the peak MAPK signal; these apoptotic cells usually contain a single nucleus. Vertical arrows in each panel indicate the positions of binucleate apoptotic cells. The insets associated with each primary panel show that the cell of interest contains two nuclei (double arrow), and apoptosis is indicated by chromatin condensation, the lack of a ring channel (rc) and by concentrated actin at the cell periphery. The two nuclei in panel A are visible in the orthogonal focal plane shown in panel A”. Bar = 20 microns (A-F), 5 microns (A’-F’).
(TIF)

S8 Fig Cytological differences between binucleate germ cell with dividing germ cells. (A) Field of post-mitotic germ cells containing a binucleate cell (double arrow); panel A’ shows a higher magnification of the binucleate cell. The two nuclei in the binucleate cell are visible in the DAPI channel (blue) and the nuclear envelope channel (red, anti-NPP-9) and are contained within a single plasma membrane (red, F-actin). The nuclear envelopes in the binucleate cell stain with similar intensity as surrounding cells with single nuclei, except for a relatively intense signal where the two nuclei are juxtaposed (arrow). The nuclear envelopes in all cells appear to have small discontinuities because of P granules on the nuclear surface. (B-E) Dividing germ cells at different stages of mitosis, higher magnifications of D and E are shown in D’ and E’, respectively. Beginning in prophase, the envelope protein NPP-9 (red) concentrates in large, round foci that stain much more intensely than the envelope in interphase cells (arrowhead); panel B shows a late prophase cell, and panel E has an early prophase cell (asterisk) below and to the right of the telophase cell. The intensely-stained clusters of NPP-9 begin to break up after cell division, as the nuclear envelopes reassemble in the daughter cells. Thus, the NPP-9 pattern distinguishes the two nuclei in anaphase or telophase cells from the two nuclei in a post-mitotic, binucleate cell. Bars = 2.5 microns.
(TIF)

S9 Fig Core expansion in the germline syncytium. (A) Wild-type, adult male gonad showing the mitotic and transition zone (TZ) regions; the panel at right shows a 3D rendered view of the core from the complete optical stack. Note that the diameter of the core (paired arrows) shows little or no expansion before, in, or after the TZ. (B) Wild-type hermaphrodite gonads from the L4 stage to the 72-hr adult. Similar to the male gonad, the core of the L4 gonad shows little expansion before, in, or after the TZ. By contrast, there is a large expansion of the core diameter in adults, and the morphology of the core in the mitotic and TZ regions increases markedly in complexity with adult age. See S7 Video for rotated, 3D views of the core. (C) Example of the variation in core morphology in a 72-hr adult. The arrow indicates a large lobe in the core. Bar = 20 microns (A-C).
(TIF)

S10 Fig Cellular anatomy of a fold. Each of the three sets of panels (A), (B-D), and (E-I) examine cell contacts in or around regions that we interpret as folds in the gonad syncytium. (A) Germ cell contacts in a wild-type gonad at 24 hours. The image shows an optical plane through the center of a gonad. The core forms a continuous, open channel through the gonad, and expands in diameter as cells move distal to proximal through the transition zone (TZ). In the expanded region the core resembles a smooth and uniform cylinder; germ cells have a simple, layer-like organization; all germ cells contact the core, through ring channels, and contact the periphery of the syncytium (here covered by sheath cells). By contrast, the core in the mitotic and TZ regions appears convoluted. Although most germ cells here are localized to the periphery of the gonad, others appear to be displaced toward the interior of the core (arrow). Panel A’ shows a higher magnification of peripheral cells (1 and 2) and interior cells (3 and 4). All four cells have apical surfaces that contact the core (HIM-4) and that contain ring channels. The basal surfaces of the peripheral cells are in direct contact with the basal lamina, because sheath cells are not present in this region (see Fig 1A and 1D). However, the basolateral surfaces of the interior cells 3 and 4 do not contact the basal lamina. (B-D) Germ cell contacts in a wild-type gonad at 48 hours. Panel B shows an optical section through the center of the gonad, and panel B’ shows a focal plane 3 microns above the plane in panel B. This gonad contains several small folds (arrow) similar to those in the younger gonad in panel A. Although groups of interior germ cells appear to occlude or disrupt the core (arrowhead in panel B), inspection of other focal planes shows that the core always remains open and continuous (arrowhead in panel B’). Panel B” shows a 3D rendered view of the core in this gonad; the numerous small holes on the surface of the core are ring channels, and rotated views of the core show that it is twisted helically (S7 Video). Comparison of the 3D core in panel B” with the optical slice shown in panel B’ shows that apparent depressions in the core (asterisks) represent infolded regions of the germline syncytium. Panel C shows a higher magnification of one of the infolded regions, and panel D shows the same region but at a lower focal plane. The interior cells 3 and 4 contact the core and have ring channels. The peripheral cells 1 and 2 appear to lack contact with the core in panel C, but contact with the core is evident in panel D. Peripheral cells 1 and 2 contact the basal lamina, but interior cells 3 and 4 show no contact with the basal lamina at any focal plane. (E-I) Panel E shows a longitudinal optical section of a gonad that appears to contain an incompletely resolved fold (open arrow) in the mid-pachytene region; note that the cell labeled 1 appears elongated or stretched in the radial direct. Panel E’ shows a 3D rendered view of the gonad core, with the ring channel of cell 1 indicated. Panels F and G show orthogonal views through the fold (panel F) and proximal to the fold (panel G) at the positions indicated in panel E; image orientations are diagrammed in panel I. The interior cell labeled 2 and the peripheral cell labeled 3 both appear to lack contact with the core in panel F, but contact with the core is evident for both cells in a third orthogonal plane shown in panel H. In similar analyses of >50 interior and peripheral germ cells, all germ cells contacted the core, but interior cells lacked contact with the periphery. These patterns of cell contact suggest that the interior cells represent infolds in the nuclear layer of the syncytium, and that core expansion results from the eversion of the folds. Bars = 5 microns (A-D).
(TIF)

S11 Fig Folds and remnants. (A) Gonads from mated, wild-type adults at 72 hrs. In 24 hr and 48 hr adults, most folds are cleared near or before (distal to) the region indicated by the vertical arrow on the top gonad. By 72 hrs, some folds remain in more proximal regions of the gonad. The folds marked with vertical arrows contain numerous germ cells (nuclei and associated membranes), while more proximal folds (open arrows) have membranous material but few or no nuclei. Many of the germ cells in the proximalmost folds appear to be undergoing apoptosis, as in the bottom gonad (long arrow); the DAPI channel of this gonad is shown in panel A’. We presume that apoptotic cells accumulate in this region because they do not contact the phagocytic sheath cells at the gonad periphery. (B) Gonads from mated, *ced-3(n3692)* adults at 72 hrs. Compared with the wild-type gonads, these gonads contain many more proximal folds with abundant nuclei (vertical arrows).
(TIF)

S12 Fig Positions of binucleate and trinucleate germ cells near folds in the germline syncytium. Each drawing represents a single optical plane of a region of the gonad containing one or more folds or remnants. The positions of all binucleate and trinucleate cells in the entire optical stack are projected onto the drawing, as described for Fig 13G and 13H. Panel A shows mated wild-type gonads at 48 hrs, and panel B shows both mated and non-mated *ced-3(n3692)* gonads at 48 hrs. The enrichment of multinucleate cells is most apparent near persistent, proximal folds in *ced-3* gonads, where they are not removed by apoptosis.
(TIF)

S1 Video PGL-1::GFP loss and cell shrinkage during apoptosis. The video shows a region just before (distal to) the gonad loop in a *ced-1(e1735)* animal as described for Fig 2A. Three cells (indicated by X) undergo apoptosis during the video. PGL-1 begins to diminish in the middle cell at 14’-18’, and shrinkage is evident by 24’. PGL-1 begins to diminish in the top left cell between 18’-20’, and shrinkage is evident by 22’. PGL-1 begins to diminish in the top right cell at 28’-30’, and shrinkage is evident by 30’. Fluorescent reporters: JJ2100 + OD70.
(MOV)

S2 Video Shrinking of an enlarged oogonium during apoptosis. The video shows two apoptotic cells (X) near the loop region of a wild-type gonad, where oogonia rapidly expand in size (see diagram in Fig 1A). The gonad expresses a reporter for germ cell membranes (red), and a reporter for sheath cells (green, CED-1::GFP). As the video begins, a second window opens at bottom left to show only the sheath signal associated with the apoptotic cells. The top apoptotic cell already has completed shrinkage, and is largely engulfed by 6 minutes. The apoptotic cell at right has expanded considerably, but then shrinks and is engulfed during the recording. Note that shrinkage of the latter cell is nearly complete before engulfment. Fluorescent reporters: JJ2575 + OD70.
(MOV)

S3 Video Mitochondria exit during apoptosis. The video shows the loop region of a wild-type gonad as in Fig 1A. The gonad expresses reporters for the plasma membrane (red) and mitochondria (green; COX-4::GFP); the plasma membrane was imaged once every third timepoint to avoid photobleaching. The first segment of the video shows the entire loop region. For size reference, a shrunken apoptotic cell (X) that lacks mitochondria is labeled in the first frame; this cell breaks down at 40 mins. The second segment replays the same video, but with a magnified view of apoptotic cells numbered 1–4. Cell 1 has largely shrunken and cleared its mitochondria at t = 0 mins in the second segment, but has an open ring channel; the ring channel closes at about t = 28 mins. Cells 2, 3, and 4 have variable numbers of mitochondria at t = 0, but cells 3 and 4 show no evidence of shrinkage. Two additional cells that are four and five cells to the left of cell 3 also undergo apoptosis at later timepoints in the video. The number of apoptotic events varied considerably in recordings of different gonads; the gonad shown here contains an atypically large number of apoptotic cells, but the events depicted are similar to those seen in other gonads. Fluorescent reporters: JJ2586 + OD70.
(MOV)

S4 Video Mitochondria retention in a *klc-1* mutant. Video of a *klc-1(ok2609)* mutant gonad. The gonad expresses reporters for the plasma membrane (red) and mitochondria (green; COX-4::GFP); the plasma membrane was imaged once every third timepoint to avoid photobleaching. At t = 0 mins the apoptotic cell (X) contains numerous mitochondria, has an open ring channel, and appears similar in size to its distal neighbors. The cell shrinks and closes its ring channel by t = 18 mins, but retains numerous mitochondria. Note that some mitochondria near the ring channel exit the cell as shrinkage begins. Mitochondria in wild-type apoptotic cells stream away from the basal surface of the cell (next to the periphery of the gonad), but mitochondria remain near the basal pole in the *klc-1* apoptotic cell. Fluorescent reporters: JJ2586 + OD70.
(MOV)

S5 Video Cofilin-actin rods in an apoptotic cell. The video shows a 3D rendered view of actin (green) in a *ced-1(e1735)* gonad; the arrow points to an apoptotic cell. Staining of the rods is much more intense than staining at the plasma membrane in non-apoptotic cells. The intense, fibrous staining at the top left is myofilaments within the sheath cells.
(MOV)

S6 Video Binucleate germ cells. Optical stack through the surface layer of germ nuclei in the pachytene region of a wild-type gonad, as in Fig 8D. The gonad is stained for DNA (blue, DAPI), and immunostained for the nuclear envelope (magenta, anti-NPP-9), actin (green, anti-ACTIN), and cofilin (red, anti-UNC-60). Double arrows indicate examples of binucleate germ cells.
(MOV)

S7 Video 3D imaging of the gonad core. 3D rendered and rotated views of the gonad core in wild-type adults at 24, 48, and 72 hrs. The germline syncytium often shows a helical twist, which is most apparent in the core at 24 hrs. Note that the 48 hr core has a deep lobe (visible at the end of the rotation), corresponding to a large fold in the syncytium.
(MOV)

S8 Video Fold/remnant in a *ced-3* gonad. The video shows an optical stack through the top half of a *ced-3* gonad stained for DNA (blue, DAPI) and F-actin (red, phalloidin). The region surrounds a fold/remnant as described in Fig 13G and 13H, and S12 Fig. The first frame in the video shows the fold/remnant in the reference plane, and contains two binucleate cells (double arrows). The second frame shows the summary cartoon of the fold/remnant with the superimposed positions of binucleate (red circles) and trinucleate (blue circles) germ cells. The third frame begins a focus through the optical stack, with binucleate germ cells labeled as 2, and trinucleate cells labeled as 3.
(MOV)
