# Supplementary material for: Binucleate germ cells in Caenorhabditis elegans are removed by physiological apoptosis
Source: PLoS Genet. 2018 Jul 19;14(7):e1007417. doi: 10.1371/journal.pgen.1007417 (PMC6053125; doi:10.1371/journal.pgen.1007417)
Supplement: S1 Table — Germ cells were scored in the late-pachytene and loop regions of the gonad. A sheath vesicle enclosing an apoptotic cell was scored as a phagosome if the separation between the vesicle membrane and the plasma membrane of the apoptotic cell was 0.2 microns or less, and scored as a phagolysosome if the separation was greater than 0.2 microns (see for examples S2 Fig). The percentage of cells contained in a phagolysosome was scored relative to total germ cells, and for total engulfed germ cells (parentheses). Irregularly-shaped nuclei with deep depressions were scored as lobed; see for example S2 Fig. Cells with perforated nuclear envelopes usually appeared to be necrotic; they were typically larger than cells with dark, compacted cytoplasm, and had far less ribosomal density. (DOCX) [file pgen.1007417.s001.docx]

**S1 Table. TEM analysis of 48hr wild-type gonads and *ced-1(e1735)* gonads**

| Feature | WT  non-apoptotic  n=366 cells | WT  apoptotic  n=78 cells | *ced-1*  apoptotic  n=131 cells |
| --- | --- | --- | --- |
| sheath wrapping |  |  |  |
| - none | 97.9% | 14.1% | 97.8% |
| - partial | 2.1 | 16.7 | 2.2 |
| - engulfed | 0 | 69.2 | 0 |
| phagolysosome | 0 | 59.0 (86.8) | 0 |
| no mitochondria | 1.6 | 84.6 | 93.1 |
| lipid droplets present | nd | 41.9 | 26.7 |
| P granules visible | 97.8 | 67.9 | 5.3 |
| microfilament bundles (large) | 0 | 0 | 70.2 |
| microfilament bundles (small) | nd | 7.7 | nd |
| lobed nucleus | 0.8 | 29.5 | 9.2 |
| electron-dense and compacted nucleoplasm and cytoplasm | 0 | 23.0 | 1.5 |
| perforated nuclear envelope | 0 | 15.4 | 2.2 |
| binucleate | 0.5 | 17.9 | 11.4 |
