# Supplementary figures and images for: Binucleate germ cells in Caenorhabditis elegans are removed by physiological apoptosis
Source: PLoS Genet. 2018 Jul 19;14(7):e1007417. doi: 10.1371/journal.pgen.1007417 (PMC6053125; doi:10.1371/journal.pgen.1007417)

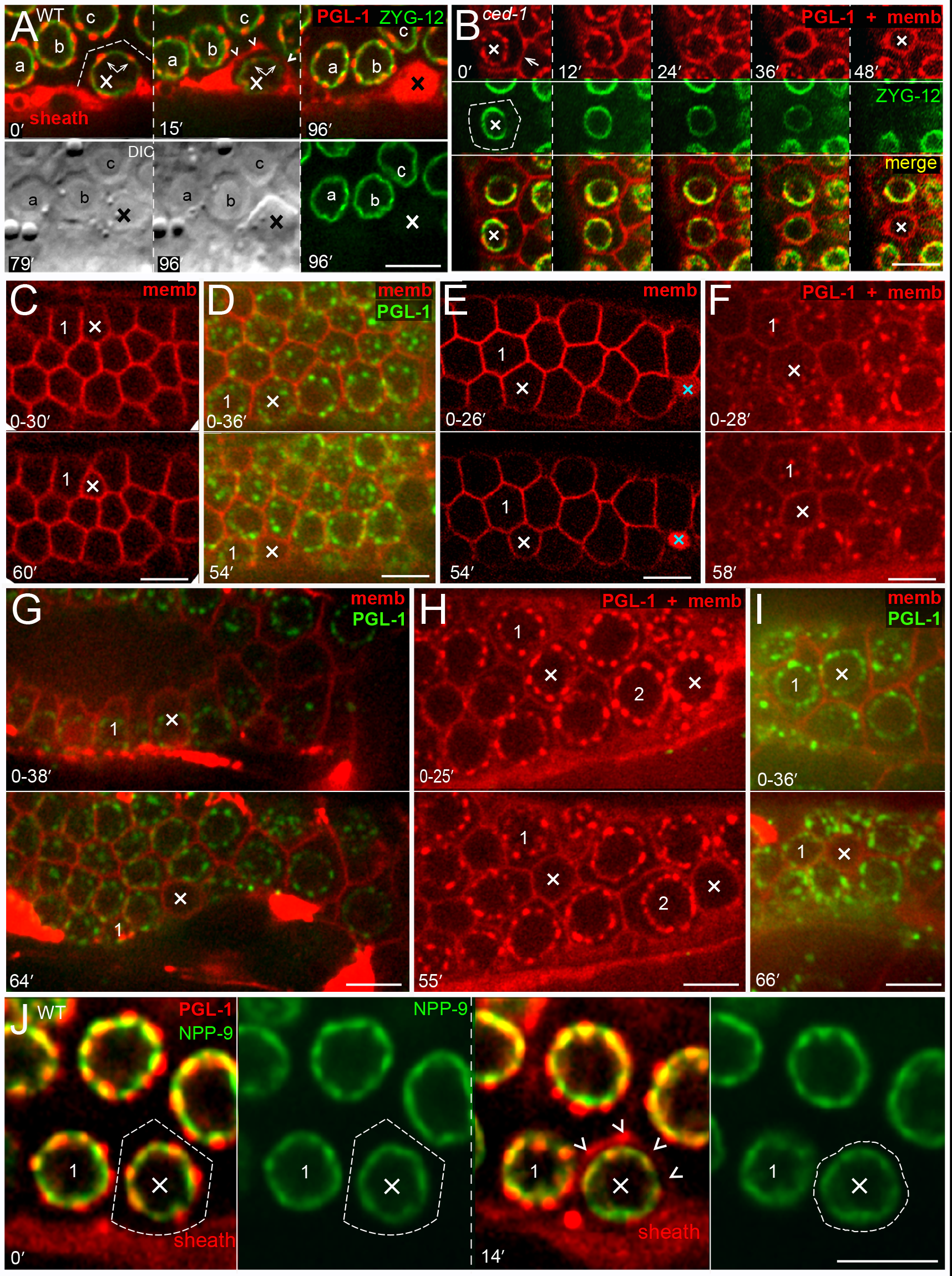

Supplement: S1 Fig — (A) Video sequence showing PGL-1 loss, cell shrinkage, engulfment, and the development of DIC refractility in a wild-type apoptotic cell (X). The gonad expresses reporters for PGL-1 (red, PGL-1::RFP) and for the nuclear envelope (green, ZYG-12::GFP). PGL-1 is expressed only in germ cells, but the PGL-1::RFP transgene used here has a heterologous promoter that drives additional, ectopic expression in the sheath cell cytoplasm. This dual expression allows the loss of PGL-1 from P granules in an apoptotic cell to be tracked simultaneously, and in the same channel, with the engulfment of that same cell by the sheath. At t = 0 mins the apoptotic cell (X, dashed outline) appears similar in size to adjacent, non-apoptotic germ cells, and has a similar level of PGL-1 on P granules (double arrow). By 15 mins, most of the PGL-1 has disappeared from the apoptotic cell, and sheath cell protrusions (arrowheads) have nearly engulfed the apoptotic cell body. The sheath protrusions outline the apoptotic cell and reveal the amount of shrinkage. The general DIC appearance of the apoptotic cell resembles that of non-apoptotic cells until about 79 mins, but the apoptotic cell becomes refractile by 96 mins as it is degraded within the sheath. (B) Video sequence of PGL-1 loss and cell shrinkage of a ced-1(e1735) apoptotic cell (X). The gonad expresses a reporter for PGL-1 (red) as above. However, because sheath cells will not engulf apoptotic cells in this mutant, a germ cell specific membrane reporter (also red) is included to track cell outlines. PGL-1 begins to diminish at about 12 mins, coincident with the start of cell shrinkage. (C-I) These recordings address whether cells destined for apoptosis differ in size from non-apoptotic cells. The sequence of panels (C-I) is arranged in a distal to proximal order through the gonad, and shows the gradual and uniform increase in cell sizes as germ cells approach the gonad loop. Each panel shows two timepoints taken from live recordings of ce [file pgen.1007417.s002.tif]

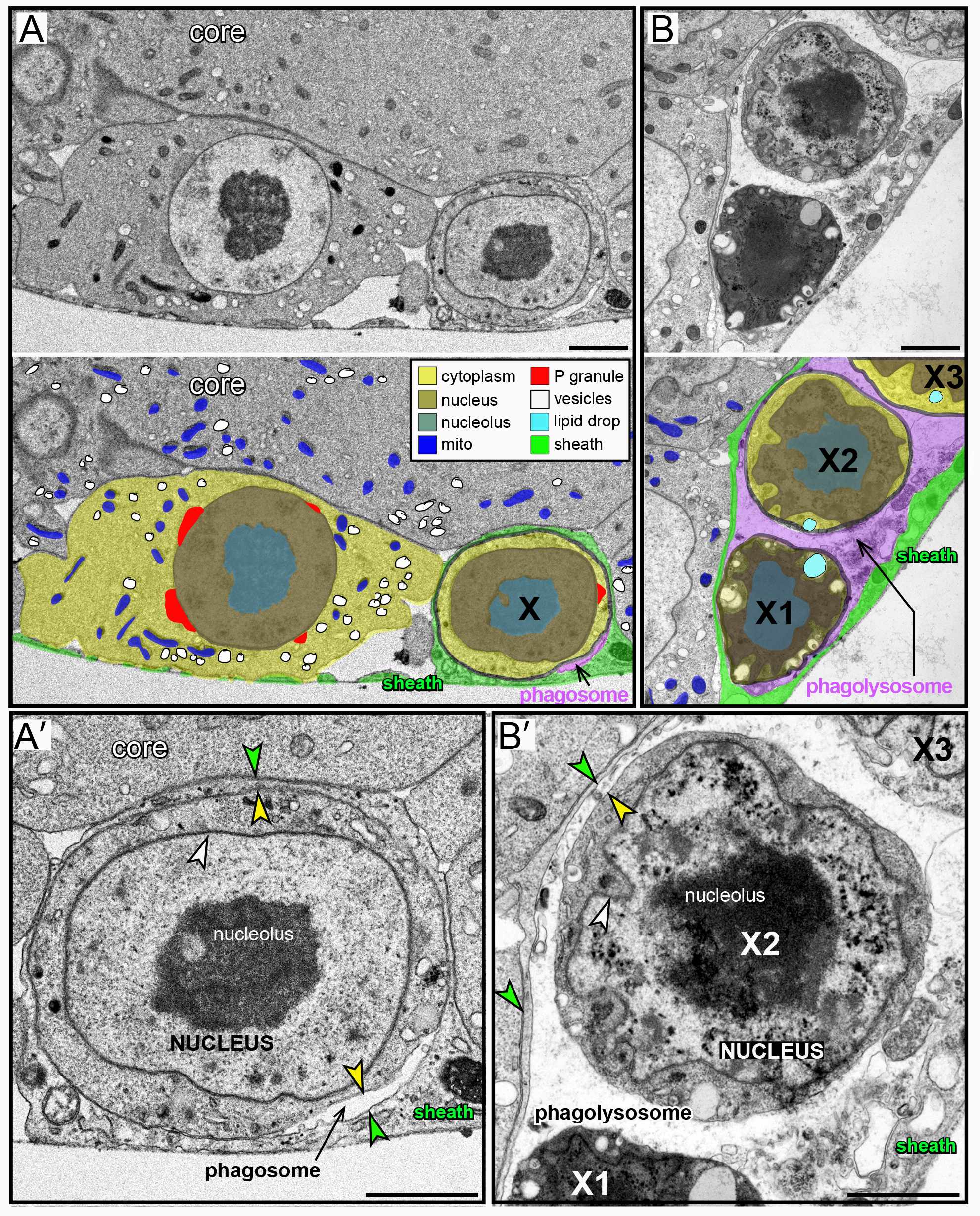

Supplement: S2 Fig — (A-A’) TEM micrograph of a wild-type gonad, comparing a normal germ cell (left) with an apoptotic cell (X). Arrowheads in panel A’ indicate the nuclear envelope (white) and plasma membrane (yellow) of the apoptotic cell, and the sheath cell process (green). The apoptotic cell has a smooth, round nucleus and has a ribosome density comparable to the non-apoptotic cell and to the core. The sheath cell has surrounded the apoptotic cell, but the sheath cavity/phagosome (purple) has not expanded into a phagolysosome. This combination of features suggests that the apoptotic cell is at a relatively early stage after shrinkage. (B-B’) The image shows three apoptotic cells (X1, X2, and X3) in a wild-type gonad; parts of two non-apoptotic cells are visible to the left of the panel. All three apoptotic cells are in a large phagolysosome (purple) within a single sheath cell. Panel B’ shows the apoptotic cells at higher magnification. Note that the densities of the cytoplasm and nucleoplasm in X2 appear greater than in the non-apoptotic cell to the left, and that the densities in X1 appear greater than in X2. Examination of semi-serial sections through this gonad showed that the X1 cell is smaller in size than the other two apoptotic cells. Bars = 1 micron. (TIF) [file pgen.1007417.s003.tif]

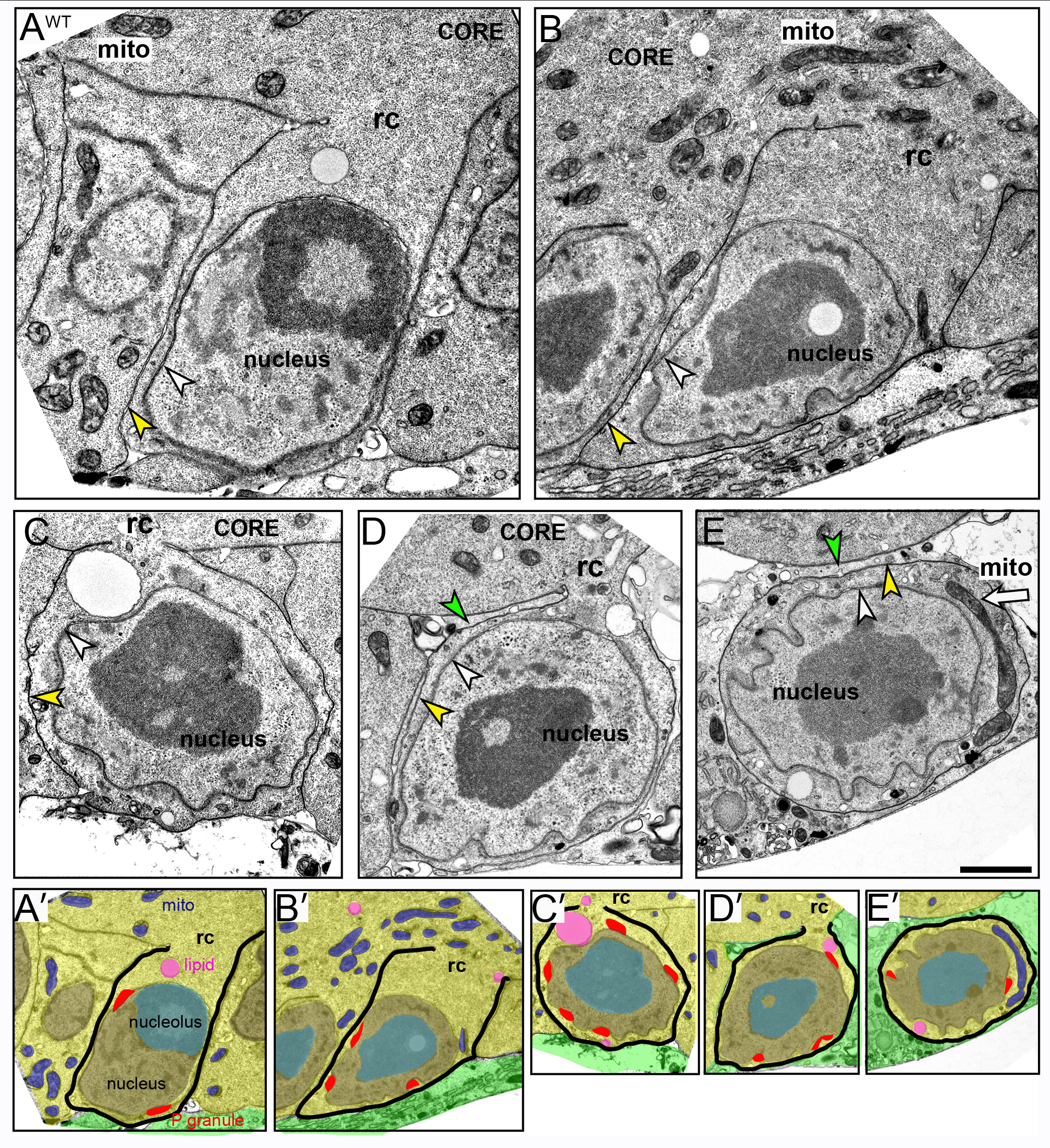

Supplement: S3 Fig — (A-D) TEM micrographs of wild-type, presumptive apoptotic, germ cells that have open ring channels but that appear to be shrinking; the cells are shown in the order of decreasing cytoplasmic volume. Arrowheads indicate the nuclear envelope (white) and the plasma membrane (yellow) of the apoptotic cell, and sheath cell processes (green) are indicated where present. Note that the cells have lost all or most of their mitochondria (cartoon at bottom, labeled as for S2 Fig). Note also that the nuclei are close to the basal pole of the cells; live imaging showed that apoptotic nuclei shift basally as they lose mitochondria. (E) Example of a closed and engulfed, wild-type apoptotic cell that retained a mitochondrion (white arrow). Bars = 1 micron (A-E). (TIF) [file pgen.1007417.s004.tif]

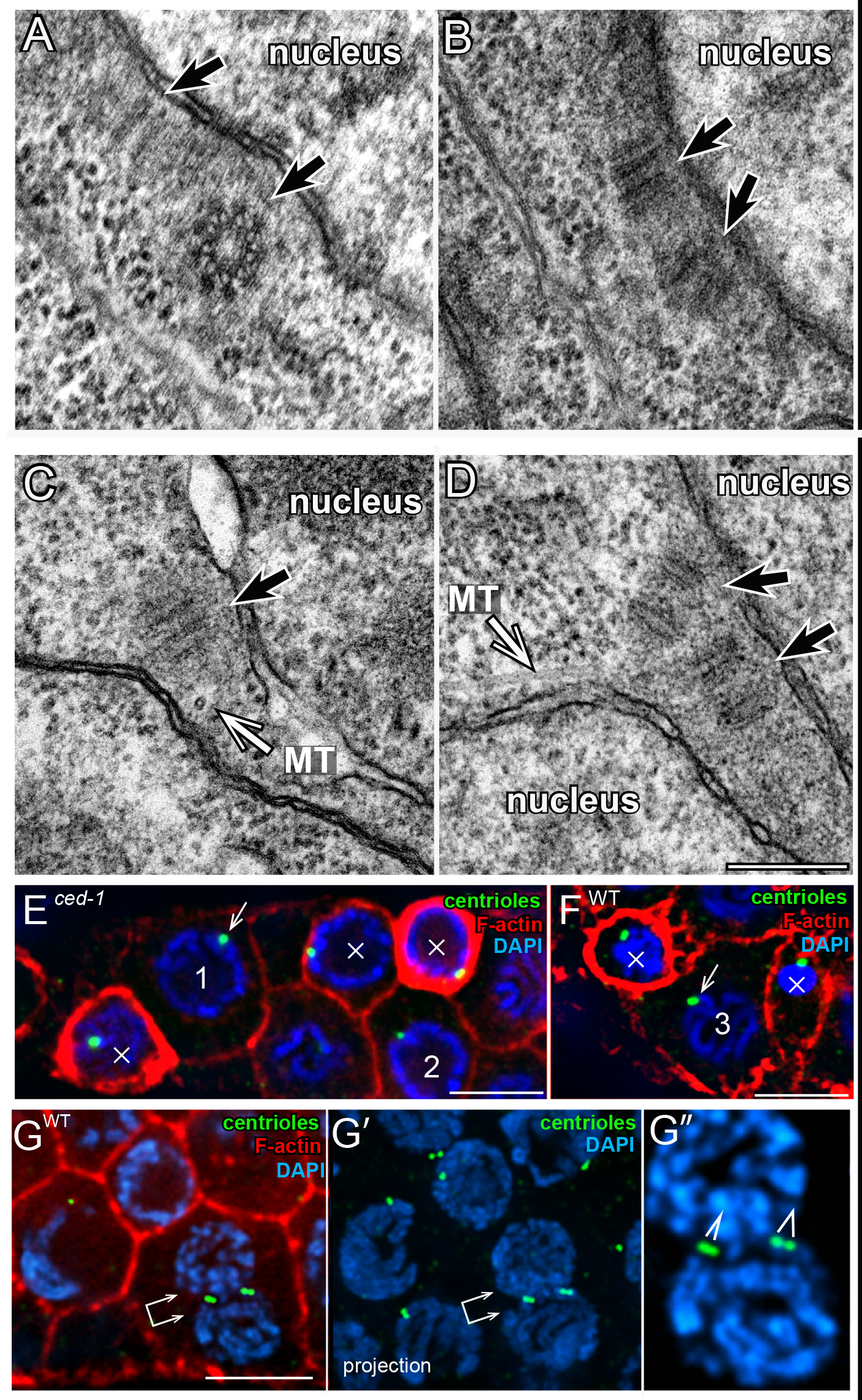

Supplement: S4 Fig — (A-D) TEM micrographs of centrioles in wild-type germ cells; panels A and B show non-apoptotic cells, and panels C and D show apoptotic cells. A centrosome consists of the paired centrioles (black arrows) and the associated pericentriolar material (PCM); the PCM typically appears in TEM micrographs as a zone of ribosome exclusion surrounding the centrioles. The paired centrioles can be aligned parallel (panel B), or orthogonally (panel A), and can have variable spacing. No MTs are visible near the centrioles in the non-apoptotic cells shown in panels A and B, but MTs (white arrows) are near the centrioles in both apoptotic cells (panels C and D); panel C shows a cross-sectional profile of an MT, and panel D shows a longitudinal profile of an MT. Note also that panel D is an example of a binucleate apoptotic cell. (E, F) Centrioles persist in apoptotic germ cells; panel E is a ced-1(e1735) gonad and panel F is a wild-type gonad. The gonads express transgenic reporters for centrioles (SPD-2::GFP in panel E and SAS-5::GFP in panels F, G) and the centrioles are visualized here by immunostaining for GFP (green). Apoptotic cells are indicated by X, and examples of non-apoptotic cells are numbered. Centrioles were observed in 19/19 apoptotic cells stained for SPD-2::GFP, and in 5/6 apoptotic cells stained for SAS-5::GFP. Panel G shows a field of non-apoptotic cells, including a non-apoptotic binucleate germ cell (double arrow). Note that the binucleate germ cell contains two pairs of centrioles (shown at higher magnification in panel G”). Panel G’ is a projection through the optical stack of field, and shows that all other germ cells contain a single pair of centrioles. Bars = 50 nm (A-D), 2.5 microns E-G. Reporters: (E and G) OC95, (F) TH61. (TIF) [file pgen.1007417.s005.tif]

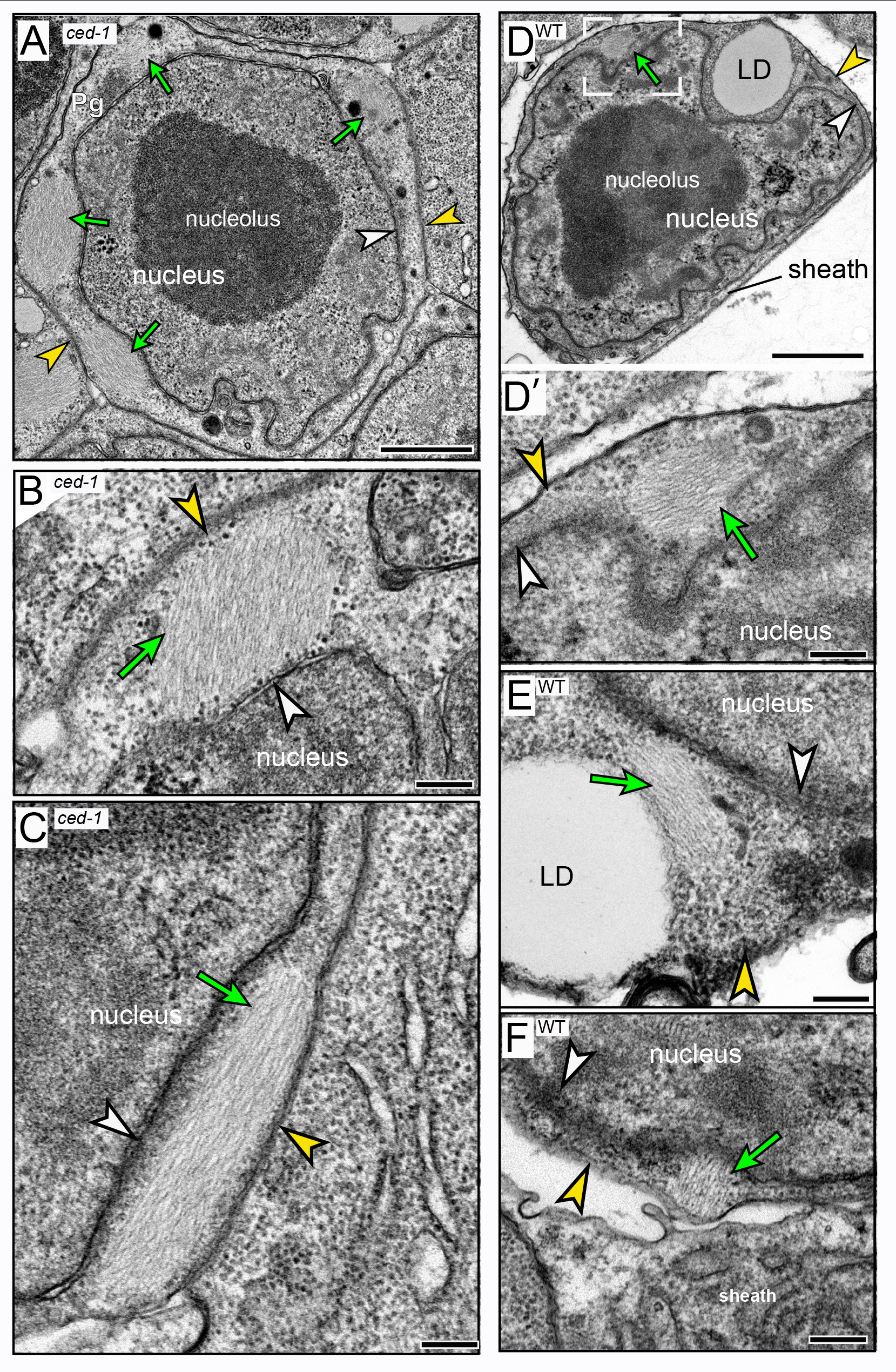

Supplement: S5 Fig — TEM micrographs showing microfilament bundles (green arrows) in ced-1(e1735) apoptotic germ cells (A-C) and in wild-type apoptotic cells (D-F). Arrowheads indicate the nuclear envelopes (white) and the plasma membranes (yellow) in the apoptotic cells. The microfilament bundles often appear to bridge the nuclear envelope and the plasma membrane, as shown in panel B. Microfilament bundles in wild-type apoptotic cells (panels D-F) are comparatively small, and appear only in cells that appear to be at advanced stages of apoptosis. Panel F shows an example of a small microfilament bundle associated with a small bump or ridge in the surface of a wild-type apoptotic cell. Bars = 1 micron (A, D), 200 nm (B, D’, E, F). (TIF) [file pgen.1007417.s006.tif]

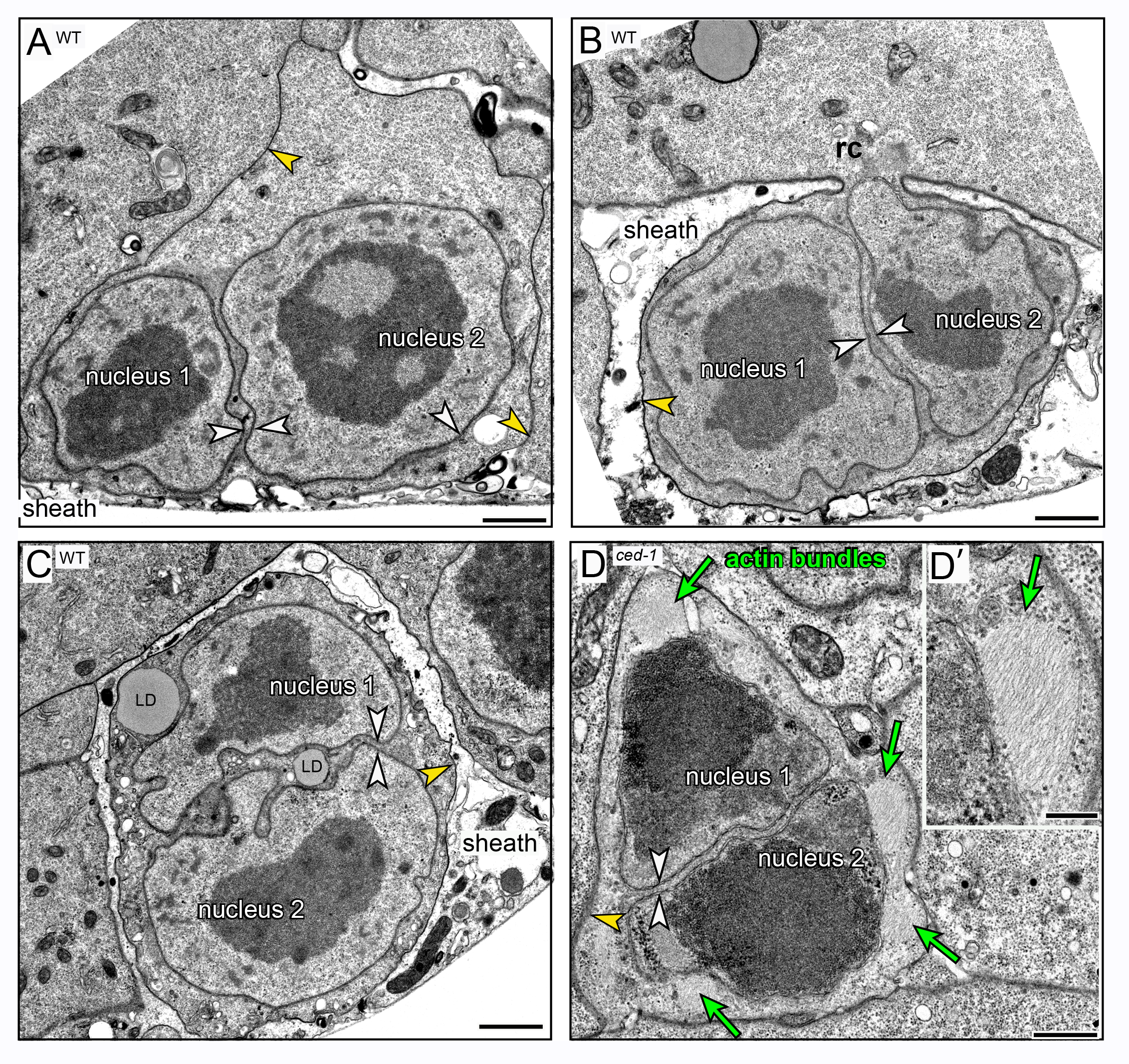

Supplement: S6 Fig — (A-D) TEM micrographs of binucleate germ cells, ordered in a developmental sequence. Arrowheads indicate the two nuclear envelopes (white) and the plasma membrane (yellow) of the binucleate cell. The binucleate cell in panel A is not engulfed and has an appreciable volume of cytoplasm, but appears to be at an early stage of apoptosis because the nuclei are shifted basally (down) and the cytoplasm appears to lack mitochondria (see text). The binucleate cell in panel B has little cytoplasm and is nearly engulfed, but retains an open ring channel (rc). The binucleate cell in panel C is fully engulfed. Panel D shows a binucleate apoptotic cell in a ced-1(e1735) gonad with several microfilament bundles, as shown at higher magnification in the inset. Bars = 1 micron A-D, 200 nm (D’). (TIF) [file pgen.1007417.s007.tif]

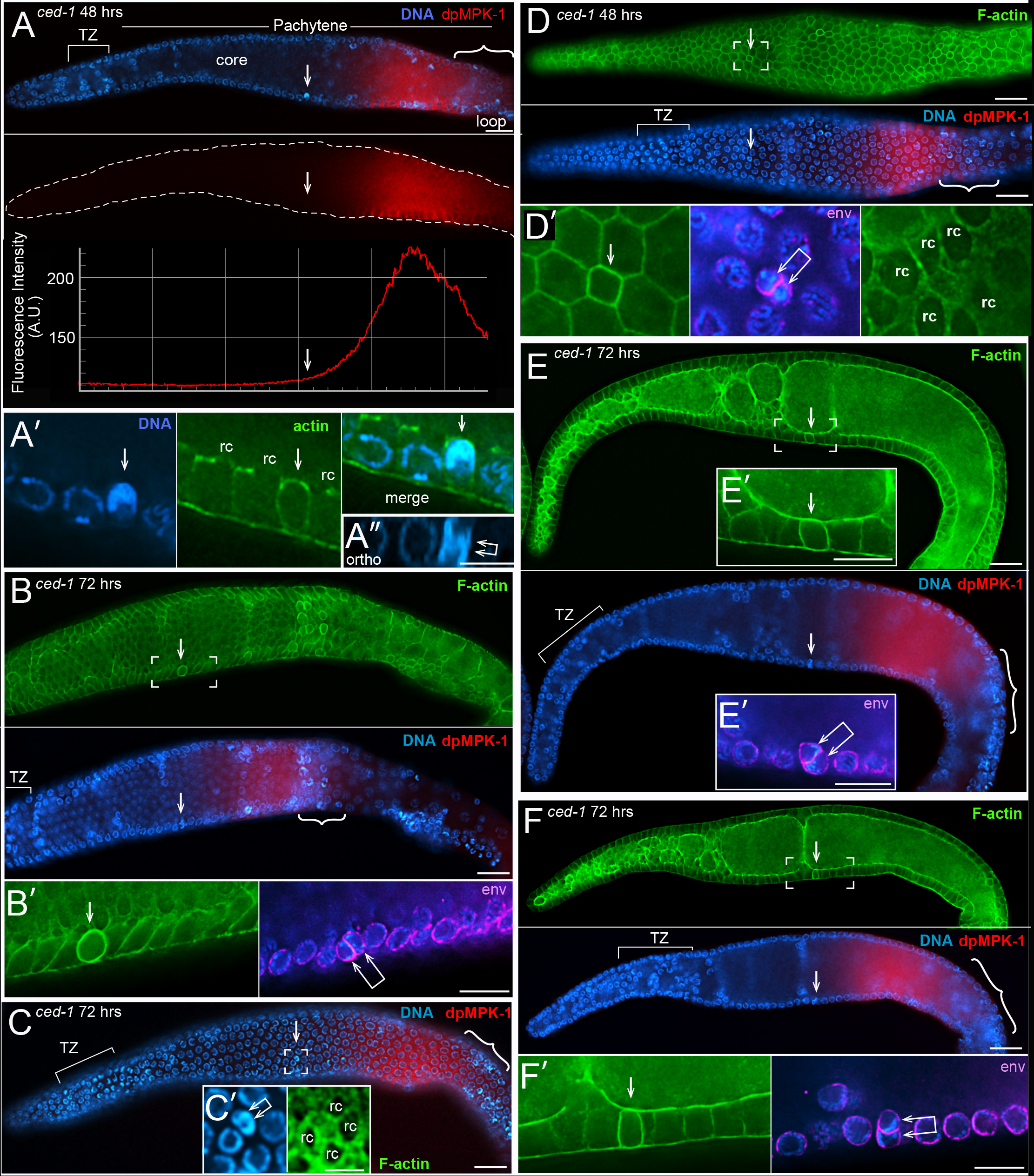

Supplement: S7 Fig — (A-F) The panels show several examples of ced-1(e1735) gonads at 48 or 72 hrs, as indicated, that were immunostained for activated MAPK (red, anti-dpMPK-1). The majority of apoptotic cells (brackets) occur after (on the proximal side of) the peak MAPK signal; these apoptotic cells usually contain a single nucleus. Vertical arrows in each panel indicate the positions of binucleate apoptotic cells. The insets associated with each primary panel show that the cell of interest contains two nuclei (double arrow), and apoptosis is indicated by chromatin condensation, the lack of a ring channel (rc) and by concentrated actin at the cell periphery. The two nuclei in panel A are visible in the orthogonal focal plane shown in panel A”. Bar = 20 microns (A-F), 5 microns (A’-F’). (TIF) [file pgen.1007417.s008.tif]

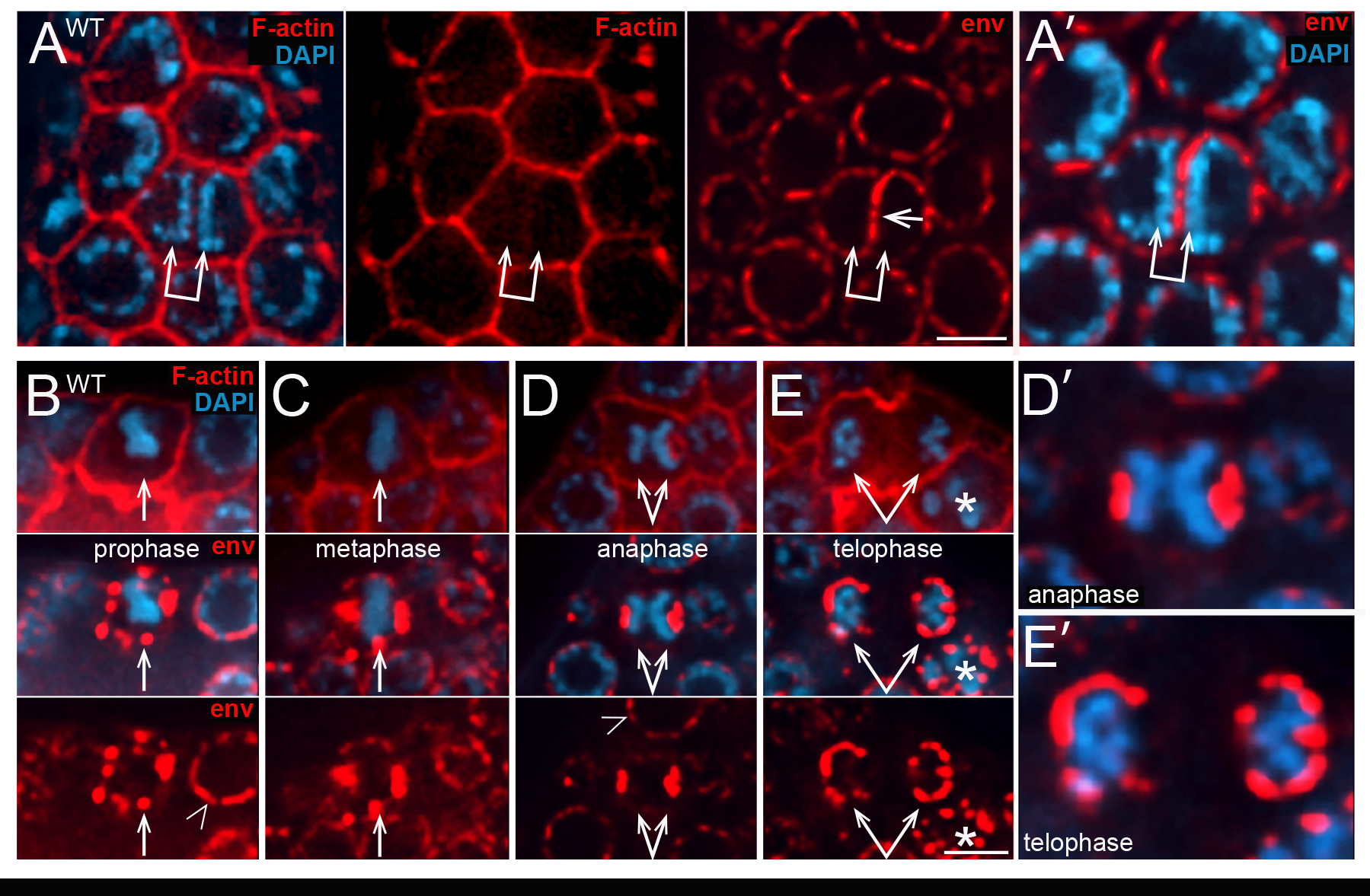

Supplement: S8 Fig — (A) Field of post-mitotic germ cells containing a binucleate cell (double arrow); panel A’ shows a higher magnification of the binucleate cell. The two nuclei in the binucleate cell are visible in the DAPI channel (blue) and the nuclear envelope channel (red, anti-NPP-9) and are contained within a single plasma membrane (red, F-actin). The nuclear envelopes in the binucleate cell stain with similar intensity as surrounding cells with single nuclei, except for a relatively intense signal where the two nuclei are juxtaposed (arrow). The nuclear envelopes in all cells appear to have small discontinuities because of P granules on the nuclear surface. (B-E) Dividing germ cells at different stages of mitosis, higher magnifications of D and E are shown in D’ and E’, respectively. Beginning in prophase, the envelope protein NPP-9 (red) concentrates in large, round foci that stain much more intensely than the envelope in interphase cells (arrowhead); panel B shows a late prophase cell, and panel E has an early prophase cell (asterisk) below and to the right of the telophase cell. The intensely-stained clusters of NPP-9 begin to break up after cell division, as the nuclear envelopes reassemble in the daughter cells. Thus, the NPP-9 pattern distinguishes the two nuclei in anaphase or telophase cells from the two nuclei in a post-mitotic, binucleate cell. Bars = 2.5 microns. (TIF) [file pgen.1007417.s009.tif]

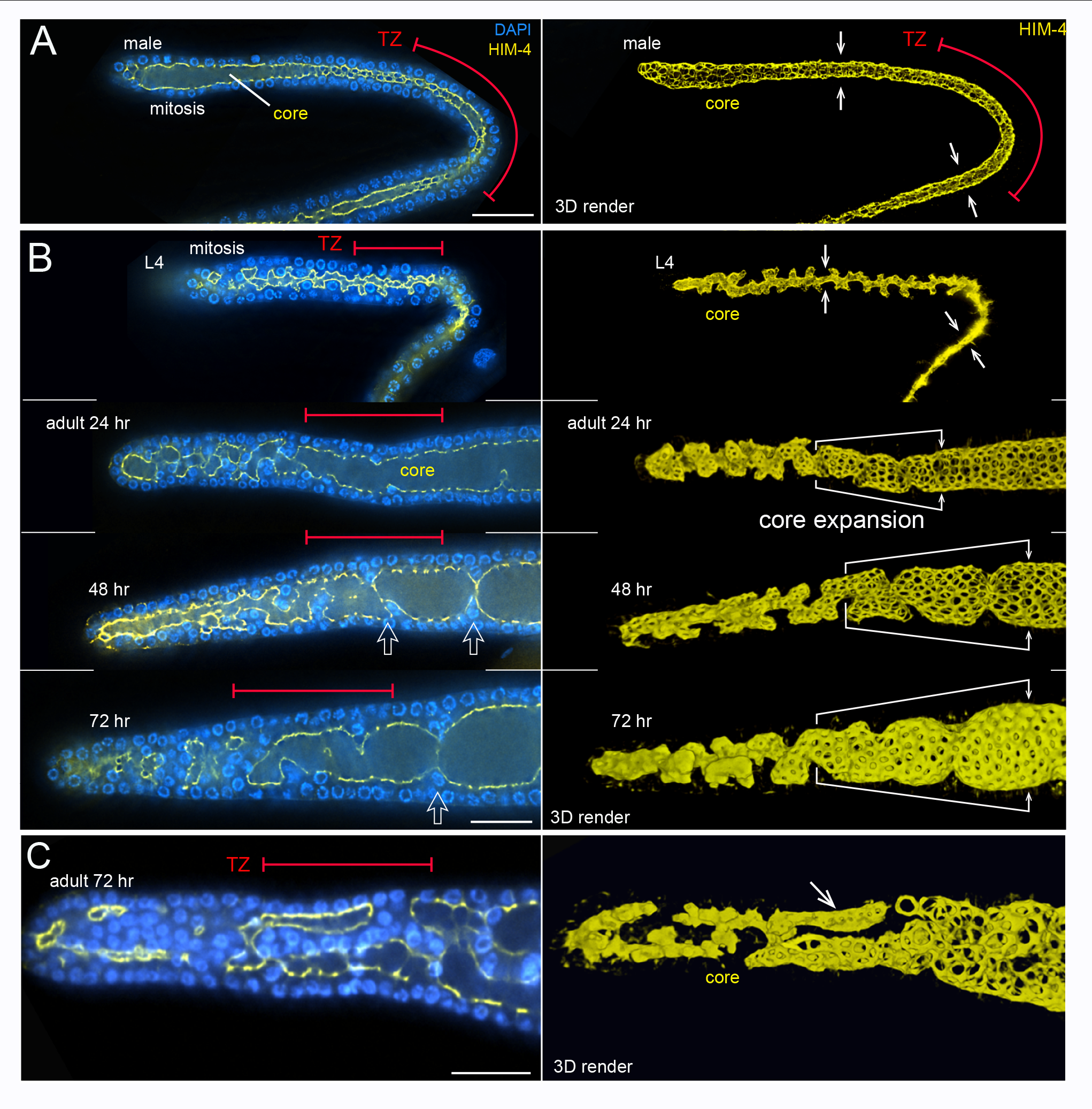

Supplement: S9 Fig — (A) Wild-type, adult male gonad showing the mitotic and transition zone (TZ) regions; the panel at right shows a 3D rendered view of the core from the complete optical stack. Note that the diameter of the core (paired arrows) shows little or no expansion before, in, or after the TZ. (B) Wild-type hermaphrodite gonads from the L4 stage to the 72-hr adult. Similar to the male gonad, the core of the L4 gonad shows little expansion before, in, or after the TZ. By contrast, there is a large expansion of the core diameter in adults, and the morphology of the core in the mitotic and TZ regions increases markedly in complexity with adult age. See S7 Video for rotated, 3D views of the core. (C) Example of the variation in core morphology in a 72-hr adult. The arrow indicates a large lobe in the core. Bar = 20 microns (A-C). (TIF) [file pgen.1007417.s010.tif]

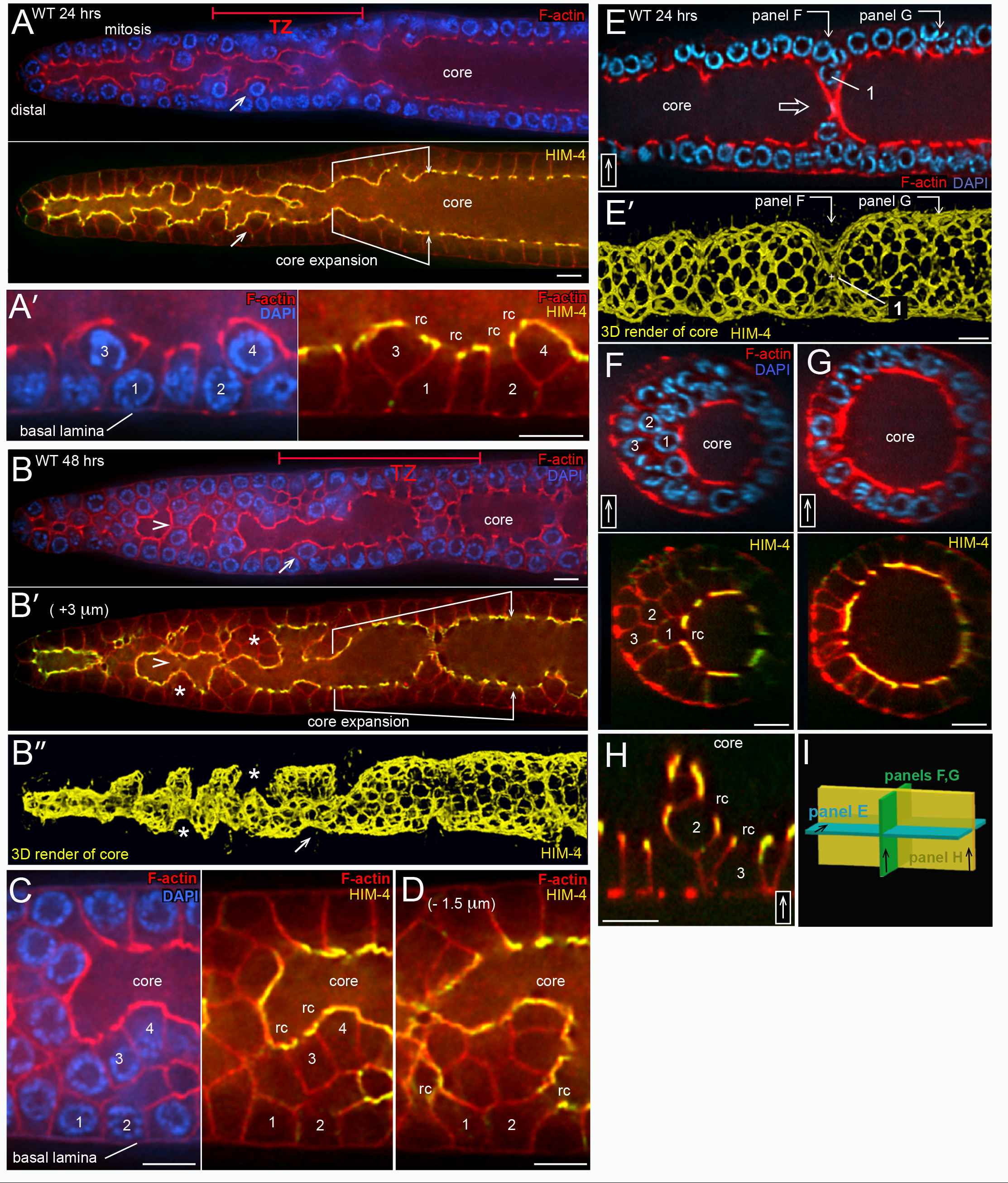

Supplement: S10 Fig — Each of the three sets of panels (A), (B-D), and (E-I) examine cell contacts in or around regions that we interpret as folds in the gonad syncytium. (A) Germ cell contacts in a wild-type gonad at 24 hours. The image shows an optical plane through the center of a gonad. The core forms a continuous, open channel through the gonad, and expands in diameter as cells move distal to proximal through the transition zone (TZ). In the expanded region the core resembles a smooth and uniform cylinder; germ cells have a simple, layer-like organization; all germ cells contact the core, through ring channels, and contact the periphery of the syncytium (here covered by sheath cells). By contrast, the core in the mitotic and TZ regions appears convoluted. Although most germ cells here are localized to the periphery of the gonad, others appear to be displaced toward the interior of the core (arrow). Panel A’ shows a higher magnification of peripheral cells (1 and 2) and interior cells (3 and 4). All four cells have apical surfaces that contact the core (HIM-4) and that contain ring channels. The basal surfaces of the peripheral cells are in direct contact with the basal lamina, because sheath cells are not present in this region (see Fig 1A and 1D). However, the basolateral surfaces of the interior cells 3 and 4 do not contact the basal lamina. (B-D) Germ cell contacts in a wild-type gonad at 48 hours. Panel B shows an optical section through the center of the gonad, and panel B’ shows a focal plane 3 microns above the plane in panel B. This gonad contains several small folds (arrow) similar to those in the younger gonad in panel A. Although groups of interior germ cells appear to occlude or disrupt the core (arrowhead in panel B), inspection of other focal planes shows that the core always remains open and continuous (arrowhead in panel B’). Panel B” shows a 3D rendered view of the core in this gonad; the numerous small holes on the surface of the core are ring channels, and rotated [file pgen.1007417.s011.tif]

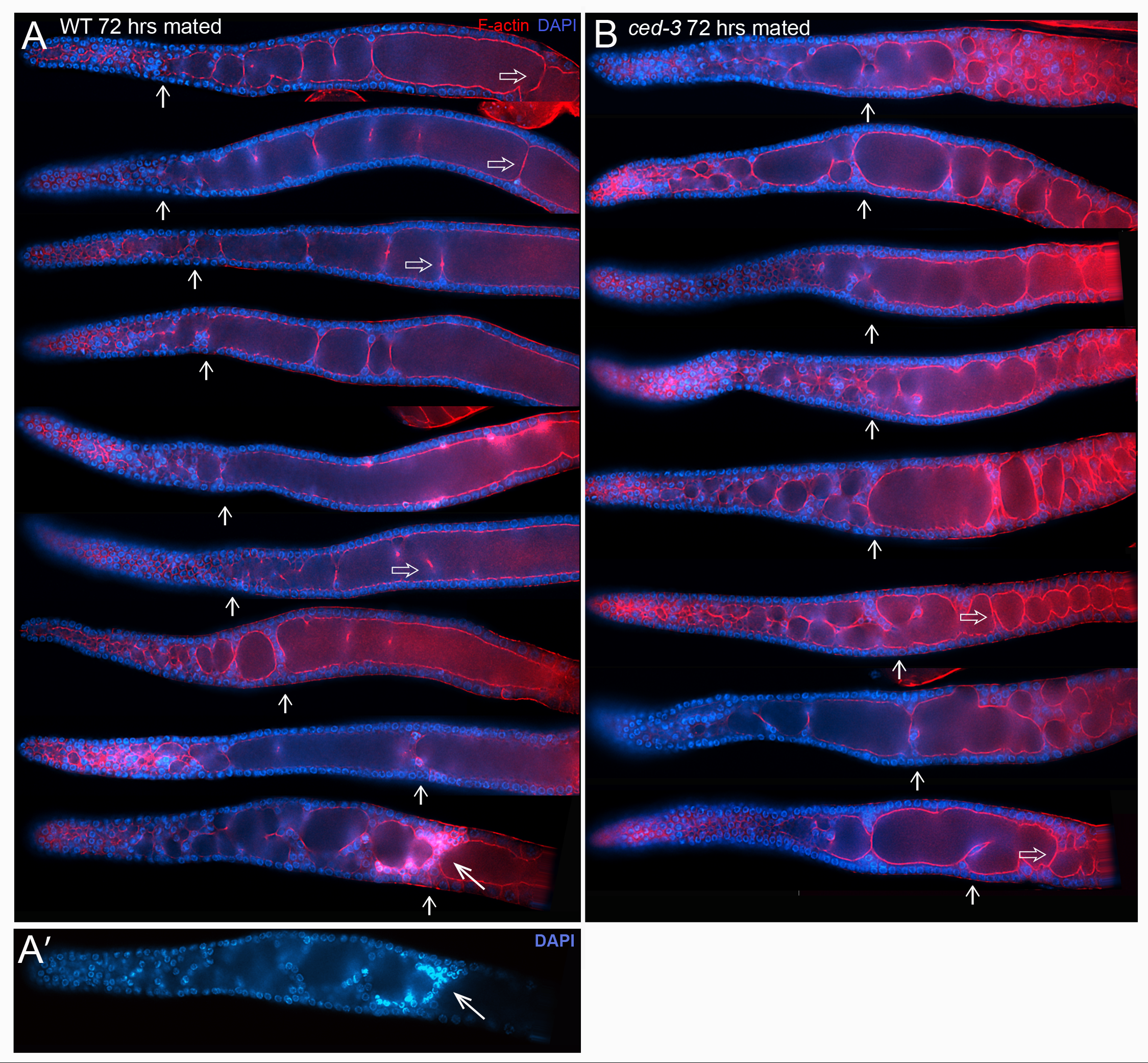

Supplement: S11 Fig — (A) Gonads from mated, wild-type adults at 72 hrs. In 24 hr and 48 hr adults, most folds are cleared near or before (distal to) the region indicated by the vertical arrow on the top gonad. By 72 hrs, some folds remain in more proximal regions of the gonad. The folds marked with vertical arrows contain numerous germ cells (nuclei and associated membranes), while more proximal folds (open arrows) have membranous material but few or no nuclei. Many of the germ cells in the proximalmost folds appear to be undergoing apoptosis, as in the bottom gonad (long arrow); the DAPI channel of this gonad is shown in panel A’. We presume that apoptotic cells accumulate in this region because they do not contact the phagocytic sheath cells at the gonad periphery. (B) Gonads from mated, ced-3(n3692) adults at 72 hrs. Compared with the wild-type gonads, these gonads contain many more proximal folds with abundant nuclei (vertical arrows). (TIF) [file pgen.1007417.s012.tif]

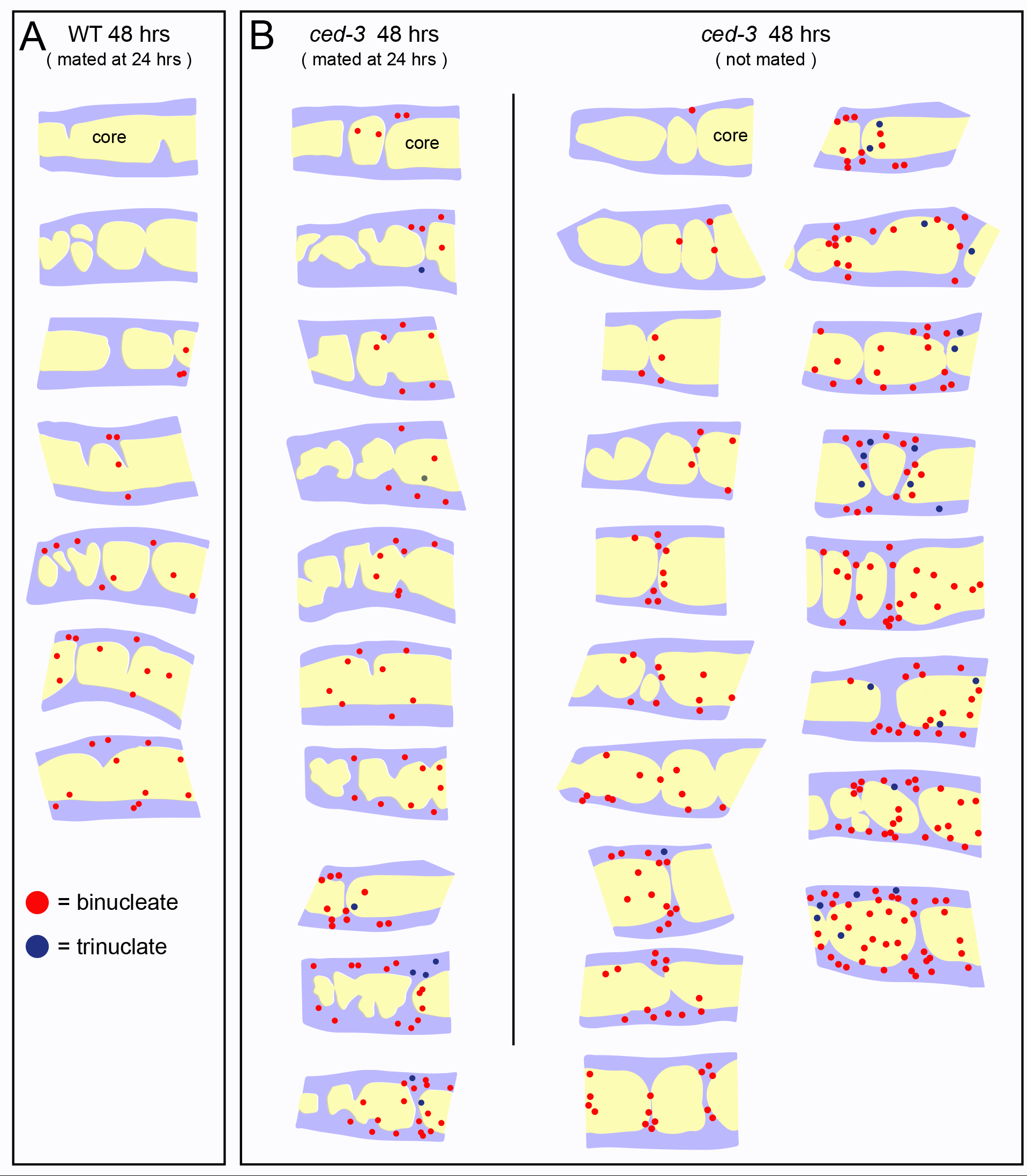

Supplement: S12 Fig — Each drawing represents a single optical plane of a region of the gonad containing one or more folds or remnants. The positions of all binucleate and trinucleate cells in the entire optical stack are projected onto the drawing, as described for Fig 13G and 13H. Panel A shows mated wild-type gonads at 48 hrs, and panel B shows both mated and non-mated ced-3(n3692) gonads at 48 hrs. The enrichment of multinucleate cells is most apparent near persistent, proximal folds in ced-3 gonads, where they are not removed by apoptosis. (TIF) [file pgen.1007417.s013.tif]
